# Neurological Effects of *Cleistocalyx nervosum* var. *paniala* Berry on Hippocampal Transcriptome, Neuritogenesis, and Synaptogenesis

**DOI:** 10.3390/nu18081200

**Published:** 2026-04-10

**Authors:** Songphon Kanlayaprasit, Worratha Parnich, Thanawin Jantheang, Pattanachat Lertpeerapan, Pawinee Panjabud, Kasidit Kasitipradit, Chayanit Poolcharoen, Thanit Saeliw, Chawanphat Muangnoi, Waluga Plaingam, Somsri Charoenkiatkul, Valerie W. Hu, Tewin Tencomnao, Tewarit Sarachana, Monruedee Sukprasansap

**Affiliations:** 1 Chulalongkorn Autism Research and Innovation Center of Excellence (ChulaACE), Department of Clinical Chemistry, Faculty of Allied Health Sciences, Chulalongkorn University, Bangkok 10330, Thailand; songphon.k@chula.ac.th (S.K.); thanit.sa@chula.ac.th (T.S.); 2 M.Sc. Program in Clinical Biochemistry and Molecular Medicine, Department of Clinical Chemistry, Faculty of Allied Health Sciences, Chulalongkorn University, Bangkok 10330, Thailand; 6570021637@student.chula.ac.th (W.P.); 6470002637@student.chula.ac.th (C.P.); 3 Ph.D. Program in Clinical Biochemistry and Molecular Medicine, Department of Clinical Chemistry, Faculty of Allied Health Sciences, Chulalongkorn University, Bangkok 10330, Thailand; 6371004037@student.chula.ac.th (T.J.); 6076654737@student.chula.ac.th (P.L.); 6176952837@student.chula.ac.th (P.P.); 6076951037@student.chula.ac.th (K.K.); 4 Biological Science and Animal Model Unit, Institute of Nutrition, Mahidol University, Nakhon Pathom 73170, Thailand; chawanphat.mua@mahidol.ac.th; 5 College of Oriental Medicine, Rangsit University, Pathum Thani 12000, Thailand; waluga.p@rsu.ac.th; 6 Institute of Nutrition, Mahidol University, Nakhon Pathom 73170, Thailand; somsri.chr@mahidol.ac.th; 7 Department of Biochemistry and Molecular Medicine, School of Medicine and Health Sciences, The George Washington University, Washington, DC 20037, USA; valhu@gwu.edu; 8 Center of Excellence on Natural Products for Neuroprotection and Anti-Ageing, Department of Clinical Chemistry, Faculty of Allied Health Sciences, Chulalongkorn University, Bangkok 10330, Thailand; tewin.t@chula.ac.th; 9 Food Toxicology Unit, Institute of Nutrition, Mahidol University, Nakhon Pathom 73170, Thailand

**Keywords:** *Cleistocalyx nervosum* var. *paniala*, sex-specific effects, neuritogenesis, synaptogenesis, transcriptome, molecular docking

## Abstract

**Background/Objectives:** Neuritogenesis and synaptogenesis support learning and cognitive function, and hippocampal neurons play central roles in these processes. *Cleistocalyx nervosum* var. *paniala* (CNP), a Southeast Asian berry, has reported neuroprotective activities, but its direct effects on hippocampal neurons remain unclear. We investigated whether CNP extract modulates hippocampal neuronal transcriptomes, neuritogenesis, and synaptogenesis. **Methods:** Primary hippocampal neurons isolated from male and female Wistar rat pups were treated with CNP extract in vitro. Cytotoxicity was assessed to define non-cytotoxic concentrations. Transcriptomic responses were profiled by RNA sequencing and validated by RT-qPCR. Neuritogenesis was quantified by neurite morphology and Sholl analysis. Synaptogenesis was evaluated by synaptic immunocytochemistry. Molecular docking of cyanidin-3-glucoside (C3G) and resveratrol was used to generate mechanistic hypotheses. **Results:** At 0.1–10 µg/mL, CNP was non-cytotoxic, whereas a 100 µg/mL dose reduced viability; therefore, 10 µg/mL was used in subsequent experiments. Exploratory RNA-seq profiling identified thousands of differentially expressed genes enriched in synapse- and neurite-related pathways, including synaptogenesis signaling, axon guidance, and neuritogenesis. RT-qPCR showed upregulation of *Igf1* in males and *Glul* in females, with sex-dependent modulation of *Bdnf* and *Cask*. CNP increased neurite length, branching, and Sholl complexity in both sexes, with a more pronounced effect in males. A male-biased effect was also observed in synapse-related marker colocalization, with increased Syn1–Psd95 colocalization detected in males. Docking suggested plausible interactions of C3G and resveratrol with regulators such as MYC, TP53, and CREB1. **Conclusions:** CNP extract alters transcriptional networks and enhances neurite outgrowth in primary hippocampal neurons in a sex-dependent manner, with male-biased effects on Syn1–Psd95 colocalization. These findings support further dose–response, mechanistic, and sex-stratified in vivo studies to evaluate its neurobiological potential.

## 1. Introduction

The hippocampus plays a central role in learning and memory by supporting neuronal growth, plasticity, and synaptic connectivity [1,2,3]. Key processes in hippocampal development and function include neurite outgrowth and synaptogenesis, which are essential for establishing and maintaining functional neural circuits. Disruption of these processes is associated with neurodevelopmental disorders, cognitive decline, and neurodegenerative diseases [4,5,6]. Therefore, agents that promote neuritogenesis and synaptogenesis may help support neuronal plasticity and brain function.

Natural products and plant-derived compounds have attracted growing interest for their potential neuroprotective and neuroenhancing effects. Polyphenolic compounds, including flavonoids and anthocyanins, have been shown to promote neurite outgrowth and synaptic plasticity through modulation of neurotrophic pathways such as NGF and BDNF signaling [7,8,9,10,11]. Thailand, with its rich biodiversity, offers numerous plant resources traditionally used to promote brain health, including *Bacopa monniera* (Brahmi), Kleeb Bua Daeng (*Nelumbo nucifera* Gaertn., *Centella asiatica* (L.) Urb., and *Piper nigrum*), and *Kaempferia parviflora* [12,13,14,15,16,17].

Among these, *Cleistocalyx nervosum* var. *paniala* (CNP), an indigenous berry native to Southeast Asia, is of particular interest. Ripe CNP fruit has traditionally been consumed as both food and folk medicine. Previous studies, including our own, have shown that CNP is rich in polyphenolic compounds, particularly cyanidin-3-glucoside (C3G) and resveratrol [18,19,20,21,22,23,24,25]. These compounds in CNP exhibit potent antioxidant, anti-inflammatory, and anti-genotoxic properties [20,23,26]. Furthermore, neuroprotective studies have previously shown that CNP extract reduces oxidative toxicity and endoplasmic reticulum stress by upregulating antioxidant enzymes and anti-apoptotic proteins in hippocampal cells [24,25], and suppresses neuroinflammation through inhibition of the MAPKs/NF-κB pathway [27,28].

Despite these promising findings, the effects of CNP extract on neuritogenesis and synaptogenesis in primary hippocampal neurons have not been investigated. Sex is also an important biological variable in hippocampal function and disease. Male and female hippocampi differ in neurogenesis, synaptic plasticity, and molecular signaling, and many hippocampal-related disorders show sex-biased prevalence or progression [29,30,31]. These differences are directly relevant to the present study because transcriptomic programs, neuritogenesis, and synaptogenesis are shaped by the underlying molecular and regulatory context of the neuron and may therefore respond differently to bioactive compounds in males and females. Our previous work on prenatal bisphenol A (BPA) exposure further supports this concept, demonstrating sex-dependent alterations in autism-related genes, transcriptional networks, and synaptogenesis in the offspring hippocampus and prefrontal cortex [32,33]. Together, these observations provide a strong rationale for examining the effects of CNP extract in sex-stratified primary hippocampal neurons.

Therefore, the aim of this study was to investigate the neuroenhancing potential of CNP extract in primary rat hippocampal neurons. We first determined a non-cytotoxic working concentration of CNP extract. We then assessed whether CNP altered transcriptomic programs using RNA sequencing, pathway and interactome analyses, and RT-qPCR validation of selected genes. To evaluate cellular phenotypes, we examined neuritogenesis using morphometry and Sholl analysis and assessed synaptogenesis via Syn1/PSD95 colocalization. Finally, we explored gene–phenotype relationships using a correlation analysis and used molecular docking to generate mechanistic hypotheses regarding potential ligand–regulator interactions. This study provides new insights into how CNP may influence neuronal plasticity and provides a basis for further investigation of CNP as a food-derived candidate relevant to neuronal plasticity and brain health.

## 2. Materials and Methods

### 2.1. Plant Material and Extraction

Ripe CNP fruits were harvested (July to September, 2020) from Lampang, Thailand, under the Plant Genetic Conservation Project under the Royal Initiative of Her Royal Highness Princess Maha Chakri Sirindhorn (RSPG). All procedures related to plant access and collection complied with Thai national regulations, particularly the Plant Variety Protection Act (1999), ensuring the necessary permits were obtained, and ethical academic standards were upheld throughout the process. The scientific name of this plant was confirmed and authenticated by Asst. Prof. Dr. Thaya Jenjittikul from the Faculty of Science, Mahidol University, Thailand. The voucher specimen of CNP was deposited at Suan Luang Rama IX Herbarium, Thailand (Code No. 9428). The extraction was performed according to the method described in our previous report [25]. The final extract was stored in the dark at −20 °C. The dark purple extract was subsequently dissolved in DMSO and filtered using a sterile polytetrafluoroethylene 0.22 μm filter to prepare a stock solution at a concentration of 100 mg/mL, which was stored at −20 °C in the dark until being used in the subsequent experiments.

### 2.2. Rat Primary Hippocampal Neurons Isolation and Culture

All animal experiments were approved by the Chulalongkorn University Animal Care and Use Committee (protocol No. 2273007). All procedures were performed in accordance with relevant guidelines and regulations, and this study is reported in accordance with the Animal Research: Reporting of In Vivo Experiments (ARRIVE) guidelines. Eight-week-old female and male outbred wild-type Wistar rats were obtained from the National Laboratory Animal Center (NLAC), Thailand, and housed at the Chulalongkorn University Laboratory Animal Center under specific pathogen-free (SPF) conditions (temperature 20–26 °C, humidity 30–70%, 12 h light/dark cycle). Food and reverse-osmosis UV-treated (RO-UV) water were provided ad libitum. Animals were clinically healthy on arrival and maintained under routine husbandry and institutional veterinary monitoring; no experimental manipulation of immune status was performed. Breeder animals and neonatal pups had no previous experimental procedures or treatments prior to tissue collection. No expected or unexpected adverse events occurred. Because the animals were used only for breeding and tissue collection (with no long-term procedures or treatments), no additional humane endpoints were defined beyond the facility’s standard monitoring and euthanasia criteria.

For sample size calculation, no power calculation was performed, and the sample size was determined pragmatically because no prior data were available. Pups were obtained from three independent litters (5–10 pups per sex per litter). Hippocampal tissues were processed separately by sex, and within each litter, hippocampi from pups of the same sex were pooled to obtain sufficient viable neurons to plate equal numbers of cells per well across all in vitro treatment conditions at a consistent density. The experimental unit was the independent litter-derived neuronal preparation for each sex (three independent litters). For all non-RNA-seq quantitative assays, the biological replicate was the independent litter-derived neuronal preparation; individual cells, dendritic segments, and technical wells were treated as nested technical observations within litter.

After birth, neonatal pups were euthanized according to the approved protocol. Pups (5–10 pups/sex/litter; 3 litters) received an intraperitoneal injection of sodium pentobarbital (100 mg/kg body weight), followed by procedures to confirm death as specified in the protocol (including an AVMA-approved physical method). Primary hippocampal cells were isolated from pups at postnatal day 1 (PND1). Brains were collected and processed separately by sex. Sex was determined by visual inspection of anogenital distance, as described by McCarthy (2015) [34].

Brains were removed and placed into a pre-chilled 15 mL conical tube containing 1× HBSS (Gibco, Waltham, MA, USA) supplemented with 30 mM glucose (Sigma-Aldrich, St. Louis, MI, USA), 2 mM HEPES (Cytiva Life Sciences, Marlborough, MA, USA), and 26 mM NaHCO_3_ (Sigma-Aldrich, St. Louis, MI, USA). Hippocampal tissues were dissected under a stereo microscope (Nikon, Shinagawa, Tokyo, Japan) as previously described [35]. Tissues were digested in 1× HBSS containing 2.5% trypsin (Invitrogen, Carlsbad, CA, USA) and 1% DNase I (Roche, Indianapolis, IN, USA) for 3–5 min at room temperature. Digestion was stopped by adding serum-containing medium consisting of Neurobasal medium (Gibco, Waltham, MA, USA), 0.5% glucose, 2 mM L-glutamine (Gibco, Waltham, MA, USA), 1% penicillin–streptomycin (HyClone, Logan, UT, USA), and 10% FBS (HyClone, Logan, UT, USA). The suspension was centrifuged at 1500 rpm for 5 min at 4 °C.

Digested tissues were resuspended in 0.05% DNase I (1 mL) and serum-containing medium (1 mL), then gently triturated into a single-cell suspension using a fire-polished glass Pasteur pipette. The suspension was filtered through a 40 µm nylon mesh and centrifuged at 1500 rpm for 5 min at 4 °C. The cell pellet was resuspended in 2 mL of maintenance medium consisting of 96% Neurobasal medium, 1× B27 (Gibco, Waltham, MA, USA), 1× L-glutamine, and 1× penicillin–streptomycin. Cells were transferred onto poly-lysine-coated 22 × 22 mm coverslips for RNA sequencing, RT–qPCR, neuritogenesis assays, and immunofluorescence staining of synaptic proteins. This study did not include in vivo treatment of pups; all treatments were applied in vitro to primary hippocampal neurons after isolation.

All pups from the three litters were used for hippocampal neuron isolation, and all litter-derived neuronal preparations were included in the experiments. Wells/coverslips were assigned to vehicle or CNP conditions according to a predefined layout established prior to plating. As treatments were prepared and applied based on this layout, group allocation was not blinded during the experiment, and outcome assessment and data analysis were performed with group labels visible. This should be considered when interpreting the morphology- and colocalization-based outcomes. No study protocol (research question, key design features, and analysis plan) was preregistered prior to the study.

### 2.3. Cell Viability Assay

The final suspension in maintenance media was plated at 10,000 cells/100 µL/well on poly-l-lysine-coated coverslips in 96-well plates. After 24 h of incubation at 37 °C, 5% CO_2_ (days in vitro 1; DIV1), cells were treated with CNP extract (0.1, 1, 10, 100 µg/mL) with a total volume of 100 µL/well (treatment day 0). For vehicle control, cells were treated with DMSO at the same volume used in the preparation of the CNP extract. The medium was half-changed every two days. After 14 days of treatment (DIV15), cell viability was assessed using CellTiter 96 AQueous One Solution (Promega, Madison, WI, USA) at 490 nm. The experiment was performed using neurons from three independent litters (biological replicates), with triplicate wells per condition for each litter-derived neuronal preparation (technical replicates).

### 2.4. RNA Extraction and RNA-Seq

Total RNAs were isolated from primary hippocampal cells treated with CNP extract or vehicle control for 14 days (DIV15) using GENEzol™ reagent according to the protocol from the company. The purity of RNAs was determined by a Nanodrop One spectrophotometer (Thermo Fisher Scientific, Waltham, MA, USA). The purified RNAs were then submitted to determine quantity and integrity using a Fragment Analyzer System (Agilent Technologies, Santa Clara, CA, USA). The results from the Fragment Analyzer were presented as the concentration, 28S:18S rRNA ratio, and RNA integrity number (RIN). The 28S:18S rRNA ratio was greater than 1.0, and the RIN was greater than 7.0 for all RNA samples. RNA samples were sent to BGI Genomics Co., Ltd., Shenzhen, China, for RNA-seq using the DNA Nanoballs Technology platform with paired-end 150 bp reads, 20 million reads per sample, and a 6 Gb stranded library, according to the company’s protocol. Briefly, mRNAs were enriched using oligo dT selection, fragmented, and converted into cDNA using random N6-primed reverse transcription, followed by second-strand cDNA synthesis. The synthesized cDNA underwent end repair and adenine addition at the 3′ end. Adaptors were then ligated to the 3′ adenylated cDNA fragments to generate the library. The library was enriched through multiple rounds of PCR amplification, and the resulting PCR products were denatured to single strands by heat. These single-stranded DNA molecules were cyclized using a splint oligo and DNA ligase to form single-stranded circular DNA nanoballs. The library was sequenced using the DNBSEQ platform. Following sequencing, the reads were filtered and subjected to quality control. Clean reads in FASTQ format were mapped to the rat reference genome Rnor_6.0 (GCF_000001895.5_Rnor_6.0) using Bowtie 2, and the gene expression levels were quantified using RSEM. Transcriptome profiles between the CNP-treated and control groups were compared using a Poisson distribution method. Genes meeting thresholds of *p*-value < 0.05 and false discovery rates (FDR) < 0.05 were considered differentially expressed for exploratory transcriptomic interpretation. The transcriptome-profiling data from this study have been deposited in the NCBI Gene Expression Omnibus (GEO) under accession number GSE284729.

### 2.5. Utilization of CNP Extract Responsive DEGs to Predict Biological Functions/Disorders, Canonical Pathways, and Interactome Network

The list of DEGs in response to CNP extract was used to overlap with the list of genes involved in each function/disorder/canonical pathway validated by Ingenuity’s Knowledge Base database using QIAGEN Ingenuity Pathway Analysis (QIAGEN Inc., Germantown, MD, USA, https://www.qiagenbioinformatics.com/products/ingenuity-pathway-analysis/, accessed on 5 December 2024) [36]. Fisher’s exact test was then performed to calculate *p*-values, and the *p*-value < 0.05 was considered statistically significant.

### 2.6. RT-qPCR Validation

RNA samples from three independent litters per group of primary hippocampal cells were reverse-transcribed into cDNA using the RevertAid First Strand cDNA Synthesis Kit per the manufacturer’s instructions. RT-qPCR was performed using three biological replicates per treatment group for both males and females. Each biological sample was run in technical triplicates. The mean Ct value from each set of technical triplicates was used to calculate the relative gene expression using the 2^−ΔΔCt^ method [37]. Statistical analyses were conducted using SPSS software version 29.0.2. Group comparisons were analyzed via a two-way ANOVA followed by a Tukey’s post hoc test. The 18S ribosomal RNA (*Rn18s*) was used as an endogenous control. Primer sequences of *Bdnf*, *Cask*, *Glul*, *Igf1*, and *Rn18s* are provided in Table S1. These four targets were selected according to three criteria: (i) they were statistically significantly differentially expressed (*p*-value and FDR < 0.05) in male or female hippocampal cells in response to CNP extract in the RNA-seq analysis; (ii) they are associated with neuritogenesis, synaptogenesis, or synaptic plasticity; and (iii) they have been reported to participate in neurodevelopmental and/or neurodegenerative processes.

### 2.7. Neuritogenesis Assay

To assess the CNP extract’s effect on neurite formation, 300 µL of cell suspension (20,000 cells/coverslip) was plated on poly-l-lysine-coated 22 × 22 mm coverslips in a 35 mm culture dish. Cells were incubated for 3–4 h at 37 °C in 5% CO_2_ for attachment, then 3 mL of maintenance medium was added for overnight recovery. On the following day (treatment day 0), cells were treated with 10 µg/mL CNP extract or vehicle control (DMSO) by replacing half of the medium with extract/DMSO-containing medium. Neurite outgrowth assays were conducted using primary hippocampal neurons derived from three independent litters, with cells from each litter divided into CNP-treated and vehicle control groups. The medium was refreshed every two days to maintain its concentration. Cultures were maintained for 14 days (DIV15), after which 80 neurons per sex and treatment were imaged across the three independent litter-derived preparations using phase-contrast microscopy. A neurite analysis was performed using the NeuronJ in ImageJ [38] to identify neurite orders, including primary and non-primary neurites. The parameters assessed included the total neurite length, average neurite length, length of primary neurites, length of non-primary neurites, number of neurite branches, number of primary neurites, and number of non-primary neurites. In addition, the Sholl analysis was performed to assess dendritic arborization by counting the number of dendritic intersections against the radial distance from the soma center [39,40].

### 2.8. Immunofluorescence for Synaptic Proteins

Primary hippocampal cells were isolated from PND1 rat pups and cultured on poly-l-lysine-coated coverslips as described above in the method for primary hippocampal cell isolation and culture. The experiment was conducted using hippocampal cells from three litters of pups per sex and treatment group. A total of 25 neurons per sex and treatment, distributed across the three independent litter-derived preparations, was used in this analysis. The application of CNP extract treatment on the primary hippocampal cells was described in the method for neuritogenesis above. Cultured neurons on treatment day 14 (DIV15) were gently washed with ice-cold PBS and fixed with freshly prepared 4% paraformaldehyde in PBS for 30 min. After fixation, the cells were washed three times with PBS for 5 min each and blocked with 3% bovine serum albumin (Capricorn Scientific, Ebsdorfergrund, Germany) at room temperature for 30 min in a dark chamber. Immunostaining was performed to detect synaptic markers, including Syn1 (presynaptic marker), Psd95 (postsynaptic marker), and Map2 (mature neuron marker). Mouse anti-Syn1 (106011; Synaptic Systems, Göttingen, Germany), rat anti-Psd95 (ab18258; Abcam, Cambridge, UK), and chicken anti-Map2 (ab5392; Abcam, Cambridge, UK) primary antibodies were added to the cells and incubated overnight at 4 °C. On the following day, the cells were incubated for 1 h at room temperature with secondary antibodies, including donkey anti-rabbit Alexa 647 (ab150063; Abcam, Cambridge, UK), donkey anti-mouse Alexa 488 (ab150109; Abcam, Cambridge, UK), and donkey anti-chicken Alexa 405 (ab175675; Abcam, Cambridge, UK). Coverslips with stained cells were mounted onto glass slides using ProLong Diamond Antifade reagent (Invitrogen, Carlsbad, CA, USA). The stained cells were imaged using an LSM800 confocal laser scanning microscope (Carl Zeiss, Oberkochen, Germany). Images were acquired using a Zeiss LSM800 confocal microscope with a 63 × oil immersion objective. All images were taken in a single optical plane under identical settings across samples. Cells with clear nuclear staining and intact neurite morphology were selected for analysis. Colocalization analysis was conducted on selected dendritic segments (approximately 100 µm from the soma) identified by Map2 positivity. For each neuron, 2–3 dendritic segments were analyzed. Colocalization analysis of the synaptic markers was performed using the Just Another Colocalization Plugin (JACoP) in ImageJ software version 1.54j release date: 12 June 2024. This analysis employed pixel-based colocalization correlation using Costes’ randomization method, with results reported as Pearson’s coefficient and percentage of colocalization between Syn1 and Psd95 markers. A higher Pearson’s coefficient indicated greater overlap between presynaptic and postsynaptic markers and was interpreted as consistent with increased synapse-related marker colocalization [41].

### 2.9. Correlation Analysis Between the Expression of Genes Response to CNP Extract and Neuro-Logical Phenotypes

The correlation analysis was performed to examine the association between the expression of selected genes differentially expressed in response to CNP extract, which were validated by RT-qPCR analysis, and the changes in neurological function, including neuritogenesis and synaptogenesis. The log_2_ (fold change) expression values of those DEGs were used to match the parameters from the neuritogenesis or synaptogenesis experiments. The Pearson’s correlation coefficient was used to calculate the correlation coefficient (R values). The R values, ranging from −1 to +1, were then used to construct a heatmap in males and females. A positive value indicates a positive correlation, while a negative value indicates a negative correlation.

### 2.10. Identification of C3G and Resveratrol in CNP Berry Extract

C3G and resveratrol were analyzed using high-performance liquid chromatography with diode-array detection (HPLC-DAD; Agilent 1260 Infinity II) with an ACE Generix C18 column. An HPLC gradient analysis was slightly modified from our previous studies [24,25,27,42]. Mobile phases for C3G were phase A (0.1% formic acid) and phase B (mixed acetonitrile, water, and formic acid); for resveratrol, phase A was 1% acetic acid in water, and phase B was acetonitrile. The flow rate was 0.7 mL/min, and 20 µL samples were injected. Detection wavelengths were 520 nm (C3G) and 270 nm (resveratrol). Sample peaks were confirmed against pure standards (≥99% purity, Sigma). Results were expressed as mg/100 g dry weight (DW).

### 2.11. In Silico Prediction of Upstream Regulator–Ligand Binding Affinity Using Molecular Docking

To evaluate whether C3G and resveratrol could interact with predicted upstream regulatory proteins associated with the DEGs, molecular docking was performed. Ligand structures (C3G and resveratrol) were retrieved from PubChem (https://pubchem.ncbi.nlm.nih.gov, accessed on 25 May 2024), and protein structures were obtained from the RCSB Protein Data Bank (PDB; https://www.rcsb.org/, accessed on 25 May 2024). Because experimentally resolved rat protein structures were not available for all targets of interest, human protein structures with suitable resolution (<3 Å; X-ray diffraction) were used for the docking analysis. Protein structures were prepared in AutoDockTools-1.5.6 by removing extraneous molecules, adding hydrogen atoms, and assigning partial charges. Docking was performed three times for each protein–ligand pair, and the average binding affinity was calculated. Protein–ligand interactions, including hydrogen bonds and other contacts, were visualized using a BIOVIA Discovery Studio Visualizer 2020 [43].

### 2.12. Statistical Analysis

For RNA-seq, differentially expressed genes (DEGs) were identified using a Poisson distribution model with thresholds of *p* < 0.05 and a false discovery rate (FDR) < 0.05. DEGs were further compared with curated gene sets associated with biological functions, disorders, and canonical pathways in QIAGEN Ingenuity Pathway Analysis (IPA), and Fisher’s exact test was applied with *p* < 0.05 considered significant. Data from RT-qPCR, neuritogenesis assays, and synaptic marker colocalization experiments are presented as mean ± SEM. These assays were performed using three independent litter-derived neuronal preparations per sex, with additional technical observations obtained within each litter. Statistical significance was assessed using a two-way ANOVA (factors: treatment and sex) followed by Tukey’s post hoc test; *p* < 0.05 was considered significant. For the cell viability assay, differences between treatment groups were analyzed using a one-way ANOVA followed by Tukey’s post hoc test.

## 3. Results

### 3.1. Effect of CNP Extract on Cell Viability in Rat Primary Hippocampal Cells

**Figure 1:**
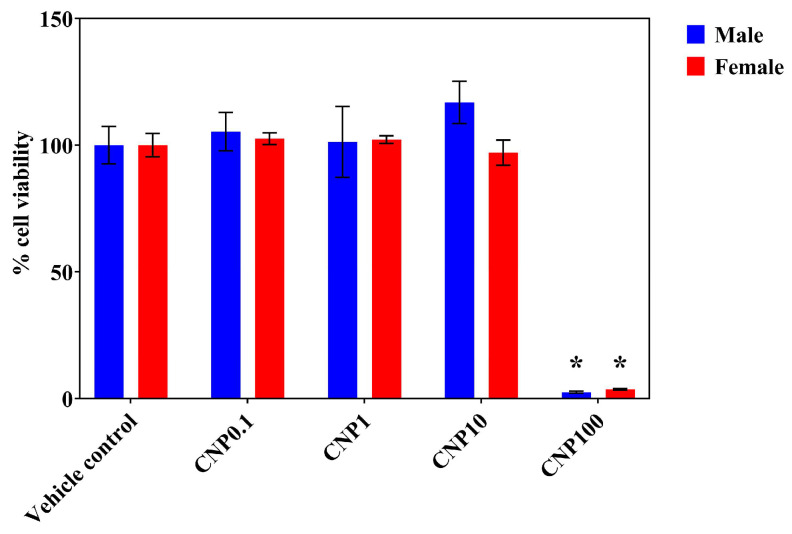
Effect of CNP berry extract on the viability of primary hippocampal cells in vitro. Primary hippocampal cells isolated from male and female rats were treated with 0.1, 1, 10, or 100 µg/mL of CNP berry extract or vehicle control for 14 days. Cell viability was assessed using MTS assays, and results are presented as the percentage of live cells. Data are presented as mean ± SEM. * *p* < 0.05 was considered statistically significant, as determined by the two-way ANOVA, followed by Tukey’s post hoc test (*n* = 3 independent litters per sex, with each litter divided into CNP0.1, CNP1, CNP10, CNP100, and vehicle control treatment groups).

We assessed the effect of CNP extract on the viability of primary hippocampal cells derived from male and female rats. Cells were exposed to 0.1, 1, 10, or 100 µg/mL of CNP for 14 days, and viability was measured at DIV15 using the MTS assay. Concentrations of 0.1–10 µg/mL did not differ from vehicle, whereas 100 µg/mL reduced viability in both sexes (Figure 1). These findings indicate that CNP was non-cytotoxic over the range of 0.1–10 µg/mL under our culture conditions. Therefore, 10 µg/mL was selected for subsequent experiments because it was the highest concentration that remained non-cytotoxic after 14 days of treatment and was used here as the working concentration for this initial exploratory mechanistic study.

### 3.2. CNP Extract Modulates Sex-Specific Transcriptome Profiles in Rat Primary Hippocampal Cells

To explore whether CNP altered transcriptional programs in a sex-dependent manner, we performed RNA-seq profiling on male and female primary hippocampal cells treated with 10 µg/mL CNP or vehicle for 14 days. We detected a total of 18,029 transcripts in male cells and 18,048 transcripts in female cells. Among these, 3155 transcripts corresponding to 2884 genes in males and 7476 transcripts corresponding to 6958 genes in females were significantly differentially expressed following exposure to CNP extract compared with vehicle control (*p* < 0.05 and false discovery rate (FDR) < 0.05). In addition, among the total DEGs identified, 695 genes were found exclusively in males, 4769 genes were found exclusively in females, and 2189 genes were common to both sexes. The full list of CNP-responsive DEGs in males and females is provided in Supplementary Table S2.

To further examine whether the sex difference in CNP-responsive DEGs might be influenced by baseline transcriptional differences, we compared the DEGs from CNP-treated males versus male controls, CNP-treated females versus female controls, and control males versus control females. This analysis showed substantial overlap between CNP-responsive DEGs and baseline male–female DEGs (Supplementary Figure S2). Specifically, 2421 male CNP-responsive DEGs and 6009 female CNP-responsive DEGs overlapped with baseline sex-differential genes, with 1952 genes shared across all three comparisons. The list of overlapping genes is provided in Supplementary Table S3. These findings indicate that a substantial proportion of genes responsive to CNP treatment were already differentially expressed between male and female hippocampal cells at baseline, suggesting that the apparent sex difference in CNP-responsive DEGs should be interpreted within pre-existing sex-specific transcriptional contexts.

### 3.3. Genes Related to Neurological Pathways and Functions Are Implicated in CNP Extract-Responsive Genes

To determine whether CNP extract-responsive genes were involved in neurological pathways and functions, we analyzed the differentially expressed genes (DEGs) using an Ingenuity Pathway Analysis (IPA). Several neurologically relevant canonical pathways were significantly enriched in both male and female cells, although the specific DEG sets differed between sexes. Notably, the synaptogenesis signaling pathway, axonal guidance signaling, neuroinflammation signaling pathway, and apoptosis signaling were among the pathways significantly associated with DEGs in both males and females (Table 1).

The IPA of neurological diseases and functions further indicated that the DEGs were associated with cognitive impairment, memory, and neuritogenesis, again with different numbers of genes involved in males and females (Table 2).

**Figure 2:**
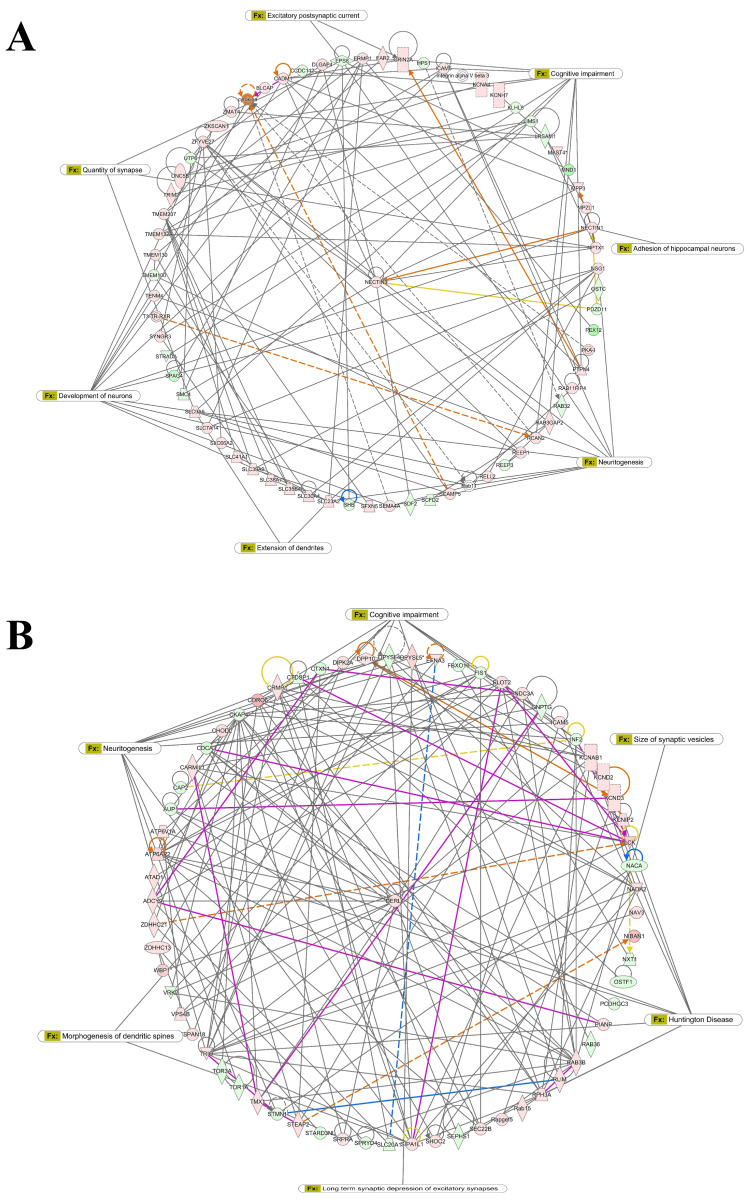
The interactome network of DEGs in rat primary hippocampal cells treated with CNP berry extracts is linked to neurological diseases/disorders and functions. IPA software predicted the gene interactome network using the list of DEGs in rat primary hippocampal cells treated with CNP berry extracts: (**A**) Male; (**B**) Female. A complete description of node and edge types is provided in the Supplementary Figure S1. * indicates that multiple identifiers in the dataset correspond to the same gene in the QIAGEN Knowledge Base.

To further examine these associations, we constructed interactome networks based on the significant DEGs in male and female cells (Figure 2). In male primary hippocampal cells, the representative network was associated with cognitive impairment, quantity of synapses, and neuritogenesis, with *Nectin3* identified as a hub gene (Figure 2A). In female hippocampal cells, the representative network was associated with cognitive impairment, Huntington’s disease, and neuritogenesis, with *Derl1* identified as a hub gene (Figure 2B). Although similar biological functions and disorders were enriched in both sexes, the specific genes represented in these networks differed. Together, these findings indicate that CNP extract modulates transcriptomic programs related to neurological functions, including neuritogenesis and synaptogenesis, in a sex-dependent manner in primary rat hippocampal cells.

### 3.4. RT-qPCR Validation Confirms Sex-Specific Regulation of Neuritogenesis- and Synaptogenesis-Related Genes by CNP Extract

**Figure 3:**
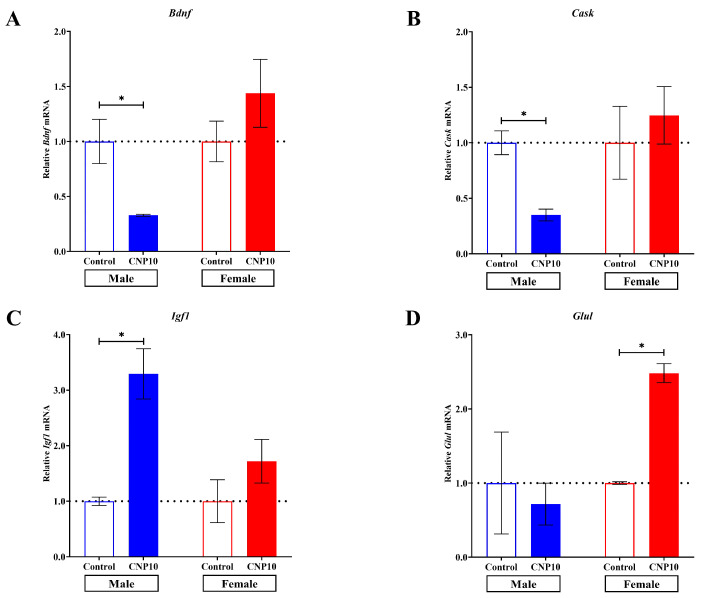
Effect of CNP extract on gene expression levels related to neurological functions, including neuritogenesis and synaptogenesis in rat primary hippocampal cells. Quantitative RT-PCR was performed to evaluate the expression levels of (**A**) *Bdnf*, (**B**) *Cask*, (**C**) *Igf1*, and (**D**) *Glul*. Data are presented as mean ± SEM. * *p*-value < 0.05 was considered statistically significant, as determined by the two-way ANOVA and Tukey’s post hoc test (*n* = 3 independent litters per sex, with each litter divided into CNP10 and vehicle control treatment groups).

Next, we validated the sex-specific expression of genes associated with neurite outgrowth and synapse formation in response to CNP extract in primary rat hippocampal cells. Four DEGs—brain-derived neurotrophic factor (*Bdnf*), calcium/calmodulin-dependent serine protein kinase (*Cask*), glutamate–ammonia ligase (*Glul*), and insulin-like growth factor 1 (*Igf1*)—were selected for the RT-qPCR analysis (Figure 3). These genes were chosen because they were significantly differentially expressed in the RNA-seq dataset (*p*-value and FDR < 0.05), are functionally linked to neuritogenesis and/or synaptogenesis, and have been implicated in neurodevelopmental or neurodegenerative disorders.

RNA samples were isolated from primary hippocampal cells treated with CNP extract, derived from three independent litters of male and female rats, and subsequently converted to cDNA for RT-qPCR. The results showed that the expression levels of *Bdnf* (Figure 3A) and *Cask* (Figure 3B) were significantly decreased in hippocampal cells from males treated with the CNP extract compared to vehicle controls (*Bdnf*: 0.33 ± 0.01 vs. 1.00 ± 0.20; *Cask*: 0.35 ± 0.05 vs. 1.00 ± 0.11). In contrast, the expression level of *Igf1* (Figure 3C) was significantly increased in males treated with CNP extract compared to vehicle controls (3.29 ± 0.45 vs. 1.00 ± 0.08). Additionally, the expression level of *Glul* (Figure 3D) was significantly increased in females treated with CNP extract compared to vehicle controls (2.48 ± 0.13 vs. 1.00 ± 0.02). Mean ± SEM values for all quantitative measures are provided in Supplementary Table S4.

These findings indicate that CNP extract regulates the expression of selected genes related to neurite outgrowth and synapse formation in a sex-specific manner.

### 3.5. CNP Extract Enhances Neurite Outgrowth and Complexity in a Sex-Dependent Manner

**Figure 4:**
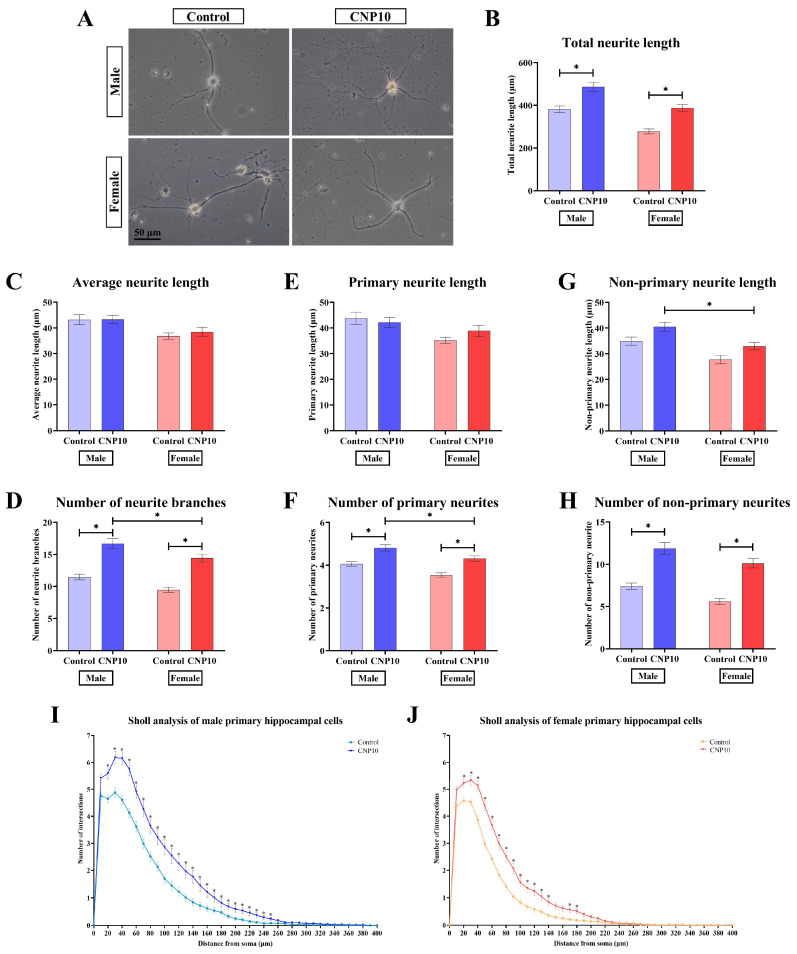
Effect of CNP extract on neurite outgrowth in male and female rat primary hippocampal cells: (**A**) Representative images of primary rat hippocampal cells treated with CNP extract or vehicle control for 14 days (scale bar = 50 µm). (**B**) Total neurite length, (**C**) average neurite length, (**D**) number of neurite branches per neuron, (**E**) primary neurite length, (**F**) number of primary neurites, (**G**) non-primary neurite length, (**H**) number of non-primary neurites, and Sholl analysis of (**I**) male and (**J**) female primary hippocampal neurons were performed to assess complexity and branching patterns. Data are presented as mean ± SEM. * *p*-value < 0.05 was considered statistically significant, as determined by two-way ANOVA followed by Tukey’s post hoc test (*n* = 80 cells per sex and treatment group, derived from 3 independent litters of neonatal rat pups).

A gene ontology analysis using IPA indicated that DEGs in response to CNP extract were associated with neuritogenesis and synaptogenesis. To determine whether the CNP extract directly affected neuritogenesis in rat primary hippocampal cells, we performed a neurite outgrowth assay using primary hippocampal neurons derived from three independent litters (Figure 4). Cells from each litter were divided into CNP-treated and vehicle control groups, and a total of 80 neurons per sex and treatment were quantified across the three independent litters.

CNP treatment significantly increased the total neurite length in both male and female hippocampal cells compared with vehicle controls (males: 486.80 ± 20.57 vs. 382.34 ± 15.18 µm; females: 388.11 ± 15.31 vs. 278.01 ± 11.86 µm) (Figure 4B). Notably, the increase in total neurite length was significantly more pronounced in males than in females. Average neurite length did not differ between groups (Figure 4C). The number of neurite branches per cell was also significantly higher in CNP-treated cells than in vehicle controls, with a more pronounced increase in males (males: 16.69 ± 0.77 vs. 11.47 ± 0.44; females: 14.44 ± 0.61 vs. 9.47 ± 0.40) (Figure 4D).

Primary neurite length was unchanged in male and female cells treated with CNP extract compared with controls (Figure 4E). However, the number of primary neurites significantly increased in CNP-treated cells of both sexes compared with controls, and male cells treated with CNP extract exhibited a higher number of primary neurites than female cells treated with the extract (males: 4.82 ± 0.15 vs. 4.06 ± 0.11; females: 4.31 ± 0.12 vs. 3.54 ± 0.11) (Figure 4F). Similarly, the length of non-primary neurites was not altered in either sex following CNP treatment, although male cells exhibited longer non-primary neurites than female cells treated with the extract (Figure 4G). In addition, the number of non-primary neurites significantly increased in CNP-treated cells of both sexes compared with controls (males: 11.87 ± 0.70 vs. 7.41 ± 0.39; females: 10.12 ± 0.56 vs. 5.59 ± 0.35) (Figure 4H).

The Sholl analysis was used to assess neurite complexity by plotting the number of neurite intersections against radial distance from the soma. This analysis revealed a significant increase in the number of intersections at radial distances of 40 µm and beyond in both male and female cells treated with CNP extract compared with vehicle controls (Figure 4I,J). Mean ± SEM values for all quantitative measures are provided in Supplementary Table S4. Together, these findings indicate that CNP extract enhances neurite outgrowth and complexity in primary hippocampal neurons, with a larger effect size in males under the present in vitro conditions.

### 3.6. Sex-Dependent Effects of CNP Extract on Syn1–Psd95 Colocalization and Synaptic Marker Distribution

**Figure 5:**
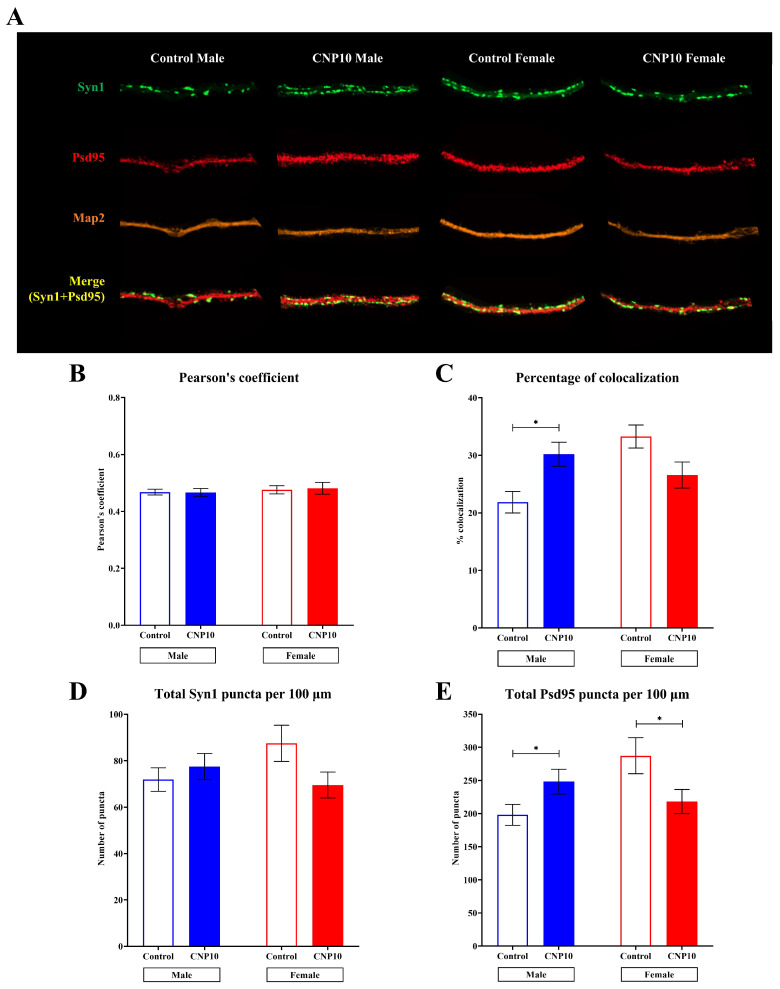
CNP extract altered Syn1–Psd95 colocalization and synaptic marker distribution in primary rat hippocampal cells in a sex-dependent manner. (**A**) Representative immunofluorescence images of synapses on primary rat hippocampal neurons treated with CNP extract or vehicle control. Images were visualized by the confocal laser scanning microscope. (**B**) Pearson’s coefficient of colocalization between Syn1 and Psd95, (**C**) percentage of colocalization between Syn1 and Psd95, (**D**) number of Syn1 puncta per 100 µm of neurite length, and (**E**) number of Psd95 puncta per 100 µm of neurite length. Data are presented as mean ± SEM. * *p*-value < 0.05 was considered statistically significant, as determined by two-way ANOVA followed by Tukey’s post hoc test (*n* = 25 cells per sex and treatment group, derived from three independent litters of neonatal rat pups).

To assess the effects of CNP extract on synaptogenesis, primary hippocampal cells were isolated and cultured for 14 days (DIV15) in the presence of CNP extract or vehicle control. Immunofluorescence staining was performed using microtubule-associated protein 2 (Map2) as a neuronal marker, Synapsin I (Syn1) as a presynaptic marker, and postsynaptic density protein 95 (Psd95) as a postsynaptic marker (Figure 5A). The relationship between Syn1 and Psd95 was evaluated using the Pearson’s correlation coefficient (Figure 5B) and the colocalization analysis (Figure 5C). The synaptic analysis relied on puncta-level colocalization on selected dendritic segments and therefore used fewer analyzable cells than the neuritogenesis assay.

**Figure 6:**
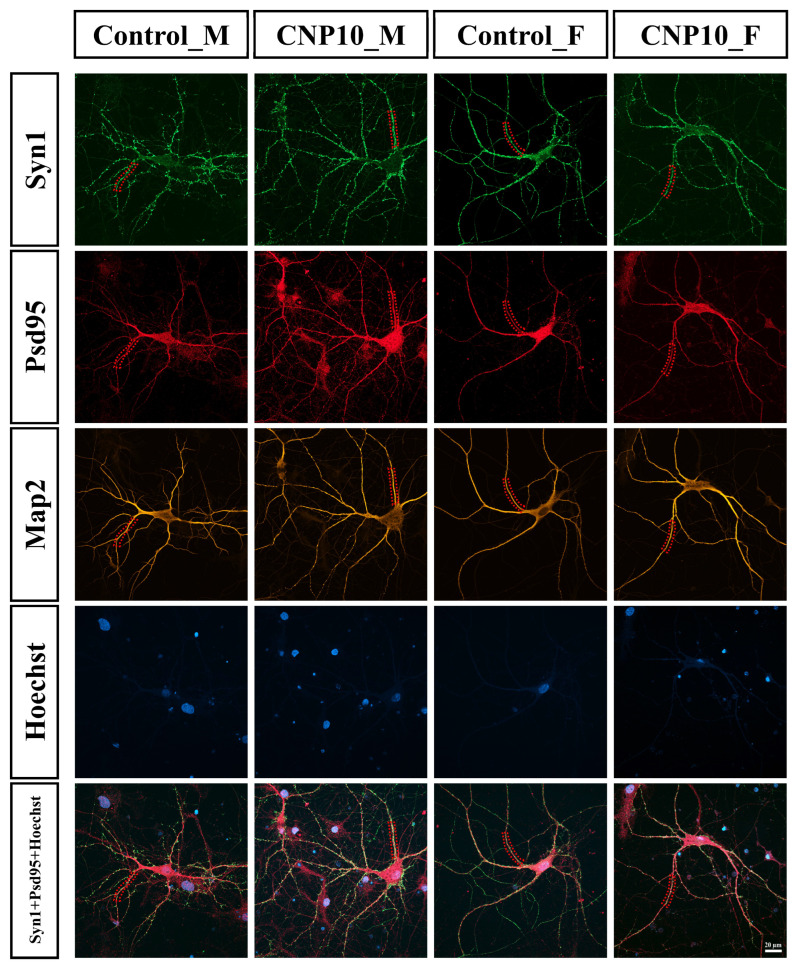
Representative full-cell immunofluorescence images of rat primary hippocampal neurons. Immunofluorescence staining was performed using microtubule-associated protein 2 (Map2) to label neuronal cell bodies and dendrites, Synapsin I (Syn1) as a presynaptic marker, and postsynaptic density protein 95 (Psd95) as a postsynaptic marker. Nuclei were counterstained with Hoechst. They were observed under the confocal laser scanning microscope (scale bar is 20 µm). The red dot indicates the region shown at higher magnification in Figure 5A.

The analysis showed positive Pearson’s correlation coefficients for the intensity distributions of Syn1 and Psd95 across all conditions, with values of r = 0.468 in male controls, r = 0.467 in male CNP-treated cells, r = 0.476 in female controls, and r = 0.482 in female CNP-treated cells. These results indicate moderate spatial alignment between Syn1 and Psd95 signals across groups. Notably, the percentage of colocalization between Syn1 and Psd95 was significantly higher in male cells treated with CNP extract than in male vehicle controls (30.18 ± 3.18 vs. 21.84 ± 2.60), whereas no such increase was observed in females (26.56 ± 3.02 vs. 33.26 ± 3.48). In addition, although the number of Syn1 puncta per 100 µm of neurite length remained unchanged (Figure 5D), the number of Psd95 puncta per 100 µm increased in males but decreased in females following CNP treatment (males: 248.20 ± 26.86 vs. 198.07 ± 23.04; females: 218.14 ± 24.57 vs. 287.33 ± 34.83) (Figure 5E). Representative full-cell images are shown in Figure 6, and mean ± SEM values for all quantitative measures are provided in Supplementary Table S4.

Together, these results indicate that although the overall spatial alignment between presynaptic and postsynaptic markers was similar across groups, the degree of Syn1–Psd95 colocalization was selectively enhanced in males treated with CNP extract. These findings support a sex-dependent effect of CNP extract on synapse-related marker colocalization, with more limited evidence for male-biased postsynaptic maturation under the present conditions.

### 3.7. Correlation Analysis Links CNP-Responsive Genes to Neurite/Synapse Phenotypes

**Figure 7:**
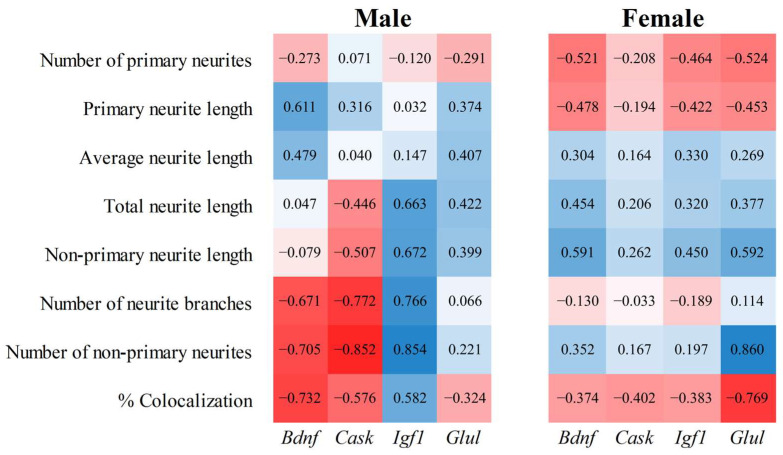
Heatmap of the correlation matrix between gene expression levels and neurological phenotypes. Correlation heatmap of the correlations between the expression levels of *Igf1*, *Bdnf*, *Cask*, and *Glul* determined by qRT-PCR and neurological functions. The color scale denotes the correlation coefficient value from red (inverse correlation) to blue (positive correlation).

To investigate whether changes in gene expression in response to CNP extract contribute to the observed enhancement in neuritogenesis and synaptogenesis, we performed a correlation analysis between neuritogenesis and synaptogenesis parameters and the expression levels (relative mRNA expression) of *Igf1*, *Bdnf*, *Cask*, and *Glul* genes. The parameters included total neurite length, primary neurite length, number of neurite branches, and % of colocalization (Figure 7).

This analysis revealed associations between gene expression and these parameters, exhibiting sex-specific patterns. An *Igf1* gene showed a strong positive correlation with the total neurite length (R = 0.663), non-primary neurite length (R = 0.672), number of non-primary neurites (R = 0.854), and number of branches (R = 0.766) in male hippocampal cells. In contrast, females exhibited moderate to poor correlations with these parameters. Additionally, the *Igf1* expression demonstrated a sex-specific correlation with synaptogenesis colocalization parameters, showing a positive correlation in males (R = 0.582) and a negative correlation in females (R = −0.383). A *Bdnf* gene demonstrated a strong positive correlation with primary neurite length and average neurite length in males (R = 0.611 and R = 0.479, respectively). However, it showed a strong inverse correlation with the number of non-primary neurites (R = −0.705) and % of colocalization (R = −0.732). Notably, sex-specific differences were observed in the correlations for primary neurite length and the number of non-primary neurites. A *Cask* gene exhibited more marked moderate to strong negative correlations in males. These included total neurite length (R = −0.446), non-primary neurite length (R = −0.507), number of non-primary neurites (R = −0.852), number of branches (R = −0.772), and % of colocalization (R = −0.576). Next, a *Glul* gene showed moderate to strong positive correlations with non-primary neurite length (R = 0.592) and the number of non-primary neurites (R = 0.860) in females. Conversely, negative correlations were observed with primary neurite length (R = −0.453), number of primary neurites (R = −0.524), and % of colocalization (R = −0.769). In males, the correlation of *Glul* expression with these parameters was weaker.

Together, these results suggest that CNP-responsive genes are associated with neuritogenesis and synaptogenesis in a sex-specific manner, highlighting potentially distinct molecular mechanisms underlying these effects.

### 3.8. Molecular Docking Analysis Reveals Sex-Specific Potential Interactions of the Main Bioactive Compounds in CNP Extract with Upstream Regulators

To explore possible molecular mechanisms through which CNP extract may modulate upstream regulators involved in transcriptional processes, we performed in silico molecular docking to assess the binding interactions between selected ligands, namely chemical compounds previously identified in CNP extract, and upstream regulator proteins. IPA identified the top 10 significant upstream regulators predicted to influence DEG regulation in male and female hippocampal cells. In males, these were Myc, Tp53, Mycn, Mlxipl, Htt, Creb1, Yap1, Ctnnb1, Fos, and Hif1a; in females, they were Tp53, Myc, Creb1, Htt, Mycn, Hnf4a, Nfe2l2, Rb1, Ctnnb1, and Tead1. Notably, Myc, Tp53, Mycn, Htt, Creb1, and Ctnnb1 were shared between the sexes.

In addition, the HPLC analysis identified the major bioactive compounds in CNP extract as anthocyanin C3G (163.25 ± 0.02 mg/100 g DW; 0.15% RSD) and resveratrol (18.42 ± 0.09 mg/100 g DW; 0.86% RSD). Details of the other phytochemicals detected in CNP are provided in Supplementary Table S5. Therefore, C3G and resveratrol were selected as representative ligands for the docking analysis.

**Figure 8:**
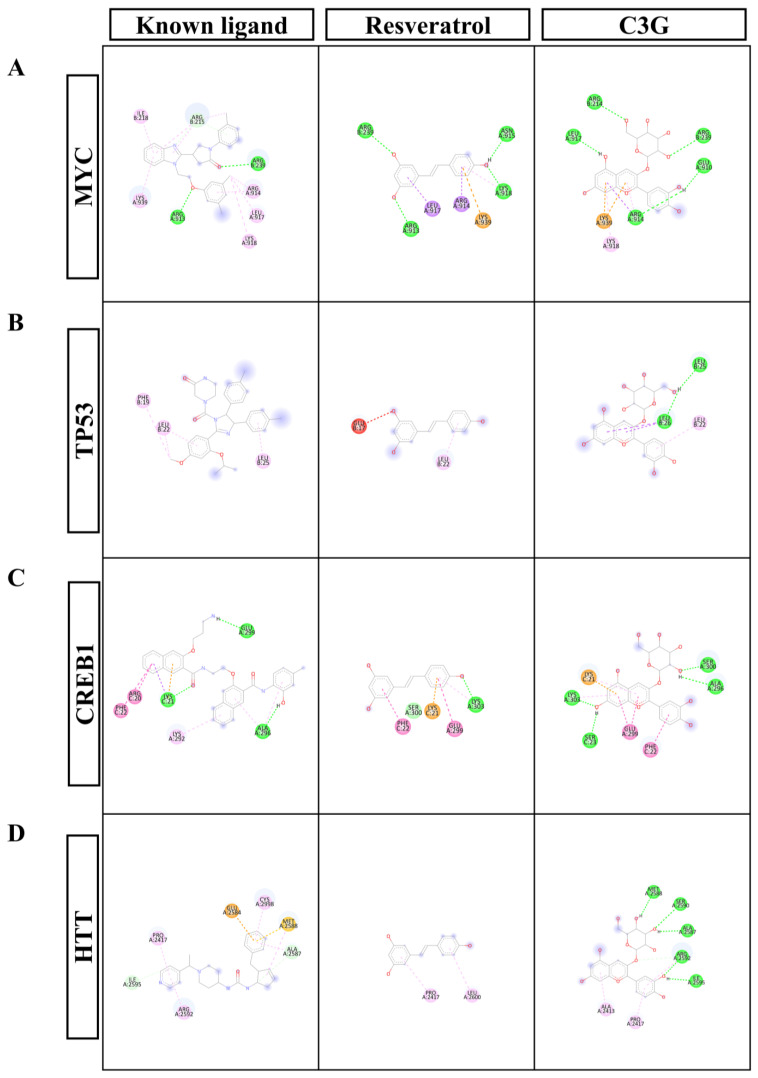
Representative 2D molecular docking interaction diagrams of selected upstream regulators with their known ligands, resveratrol, and cyanidin-3-glucoside (C3G). The (**A**) MYC, (**B**) TP53, (**C**) CREB1, and (**D**) HTT are significant upstream regulators of male or female DEGs in rat primary hippocampal cells treated with CNP berry extract. Dashed lines are color-coded to indicate interaction types between the ligand and protein residues: bright green = conventional hydrogen bond; light green = carbon–hydrogen interaction; yellow–green = π–lone-pair interaction; pink = hydrophobic/π–alkyl interaction; deep pink = π–π stacking interaction; purple = π–sigma interaction; orange = π–cation/anion interaction; yellow–orange = π–sulfur interaction; red residue circle = unfavorable interaction site; red dashed line = unfavorable contact. Pale or lightly shaded residue circles without an interaction line indicate van der Waals contacts, and a blue halo surrounding residues represents the solvent-accessible surface. Residue circles are colored according to the most favorable interaction present at that site when multiple interaction types occur along different dashed lines. The 2D interaction diagrams for the remaining regulators are shown in Supplementary Figure S3.

The docking analysis was performed to assess the binding affinity of each ligand to selected upstream regulator proteins and to identify key interacting residues within each protein–ligand complex. Representative upstream regulators shared between male and female DEGs, including Myc, Tp53, Creb1, and Htt, together with their binding to the known ligands, resveratrol, and C3G, are shown in Table 3, and the corresponding 2D interaction diagrams are presented in Figure 8A–D. Full docking results, including PDB IDs and amino acid interaction details, are provided in Supplementary Table S6. The corresponding 2D interaction diagrams for the remaining regulators are shown in Supplementary Figure S3. Overall, several upstream regulators exhibited favorable binding affinities to C3G and resveratrol relative to their known ligands, whereas others showed weaker or no effective binding. Together, these findings suggest that the major bioactive compounds in CNP extract may interact with selected upstream regulators in a sex-dependent manner and provide preliminary, hypothesis-generating mechanistic insights for future validation.

## 4. Discussion

**Figure 9:**
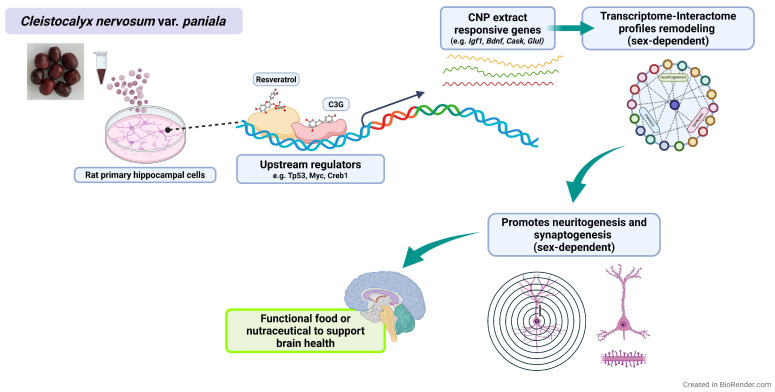
Proposed schematic mechanism of sex-dependent transcriptomic, neuritogenesis, and synaptogenesis responses to *Cleistocalyx nervosum* var. *paniala* berry in primary rat hippocampal neurons. CNP extract—rich in cyanidin-3-glucoside (C3G) and resveratrol—interfaces with upstream transcriptional regulators (e.g., Myc, Tp53, Creb1), which in turn modulate neurite- and synapse-related genes validated by qPCR (*Igf1*, *Bdnf*, *Cask*, and *Glul*) alongside additional CNP-responsive targets. The resulting sex-biased transcriptional programs remodel the transcriptome/interactome and are associated with enhanced neurite elaboration and synaptic readouts in vitro, outlining a putative pathway for CNP’s neurobiological effects (this figure was created with www.Biorender.com).

Neurodegenerative diseases are increasing as the global population ages, and natural products have attracted interest for their potential to help prevent or slow these conditions, including Alzheimer’s disease. Our previous work showed that *Cleistocalyx nervosum* var. *paniala* (CNP) extract exerts antioxidant, anti-inflammatory, and pro-survival effects in neuronal cell lines through the SIRT1/Nrf2, NF-κB, and MAPK pathways [25,27,28]. However, its effects on neuronal connectivity in primary neurons had not been examined. Here, using sex-stratified primary rat hippocampal neurons, we found that CNP promoted neuritogenesis in both sexes, enhanced synaptic colocalization mainly in males, and induced sex-dependent transcriptomic responses. Correlation analysis and molecular docking further provided supportive, hypothesis-generating insight into possible mechanisms underlying these effects. A schematic summary of the proposed sex-dependent mechanisms is shown in Figure 9.

We first established the working concentration of CNP. Consistent with our previous findings in HT22 cells [24,25], low to moderate concentrations (≤10 µg/mL) did not reduce viability, whereas 100 µg/mL was cytotoxic in both sexes. This supports careful concentration optimization because polyphenol-rich extracts can exhibit biphasic behavior, with antioxidant effects at lower concentrations and pro-oxidant effects at higher ones [47]. Similar narrow protective ranges have been reported for other berry-derived polyphenols, including resveratrol and anthocyanins [48,49]. Because this study was designed as an initial exploratory mechanistic evaluation, we selected 10 µg/mL for downstream assays because it was the highest concentration that remained non-cytotoxic after 14 days under our culture conditions. This allowed us to test whether CNP could induce measurable transcriptomic and functional effects while remaining within a safe exposure range. However, neuritogenesis, synaptogenesis, and transcriptomic outcomes were assessed at only this single concentration. Therefore, the absence of dose–response analyses is a limitation of the present study, and future studies will be needed to define the concentration dependence and optimal effective range of CNP in neuronal models.

RNA-seq showed that CNP altered gene expression in a sex-dependent manner, with female primary hippocampal cells exhibiting more differentially expressed genes (DEGs) than male cells (6958 vs. 2884), consistent with prior evidence that male and female hippocampal neurons differ in baseline gene regulation and sensitivity to perturbations [50]. Although this asymmetry could raise concern about technical bias, the RNA-seq quality metrics were comparable across samples, arguing against an obvious sequencing-quality artifact as the sole explanation. In addition, the overlap analysis showed that a substantial proportion of CNP-responsive DEGs in both sexes also overlapped with baseline male–female DEGs, indicating that many genes responsive to CNP treatment were already differentially expressed between male and female hippocampal cells under baseline conditions. These findings suggest that the sex-dependent response to CNP occurs within distinct pre-existing transcriptional contexts. At the same time, because the RNA-seq libraries were generated from pooled neurons obtained from multiple litters, pooling-related variability cannot be fully excluded.

Despite the difference in DEG counts, functional enrichment analyses in both sexes converged on related themes involving memory, neuritogenesis, synapse-related functions, and pathways such as synaptogenesis signaling and axon guidance (Table 1). IPA also linked the DEG sets to “cognitive impairment,” which does not imply that CNP induces cognitive impairment, but rather that it modulates networks previously implicated in these processes. Together, these findings support the biological rationale for including sex as a key variable in the present study. Male and female hippocampal neurons differ in molecular context and signaling responsiveness, and our results show that the CNP extract elicits distinct transcriptomic and cellular responses in the two sexes. Thus, sex in this study should not be viewed as merely a descriptive grouping factor, but as a biologically meaningful determinant of neuronal responsiveness to the CNP extract.

The interactome analysis identified different hub genes in males and females, with *Nectin3* emerging in males and *Derl1* in females. Both genes have been linked to neuronal structure and synaptic function, suggesting that CNP may influence sex-biased gene networks relevant to hippocampal connectivity. In particular, Nectin-3 has been associated with learning and memory deficits, tauopathy, stress-related disorders, and Alzheimer’s disease [51,52,53,54], whereas Derlin-1 contributes to neuritogenesis and branch formation, and *Derl1*-deficient brain tissue shows altered cholesterol biosynthesis pathways [55,56]. These findings are consistent with a role for CNP in modulating gene networks relevant to hippocampal structure and function.

At the cellular level, CNP increased total neurite length, neurite branch number, and the number of primary and non-primary neurites in primary hippocampal neurons from both sexes, with a larger effect in males, whereas primary neurite length itself was not significantly changed. In the synaptogenesis assay, CNP increased Syn1–Psd95 colocalization in males but not females, and this male-specific effect was accompanied by increased Psd95 puncta density. Because Psd95 is a key organizer of glutamate receptors and synaptic stabilization [57], this pattern is consistent with enhanced postsynaptic maturation and excitatory connectivity in males. In females, by contrast, neurite outgrowth was enhanced without a parallel increase in synaptic puncta, suggesting that structural elaboration did not translate into comparable postsynaptic strengthening under these conditions. Estrogen receptor signaling can regulate Psd95 in some contexts [58], raising the possibility that phytoestrogenic CNP components may interact differently with ER-related pathways in females.

The synaptogenesis assay used fewer cells than the neuritogenesis assay, reflecting its more stringent and lower-throughput analysis requirements. While neuritogenesis was assessed using broader morphological features, synaptogenesis required puncta-level colocalization analysis at a much smaller spatial scale, limiting the number of neurons that could be reliably quantified per condition. Therefore, the male-specific increase in Syn1–Psd95 colocalization should be interpreted cautiously. Nevertheless, this initial analysis suggested that CNP extract may influence synapse-related marker organization and postsynaptic maturation, as the effect was observed across neurons derived from three independent litters and was accompanied by increased Psd95 puncta in males. Larger independent studies will be needed to confirm the robustness of this finding. In addition, because group allocation was not blinded during experiment conduct and outcome assessment and data analysis were performed with group labels visible, observer-related bias cannot be fully excluded, particularly for morphology- and colocalization-based endpoints.

Correlation analyses suggested sex-specific relationships between CNP-responsive genes and neuronal phenotypes. In males, *Bdnf* correlated positively with primary and average neurite length but negatively with non-primary neurite number, branch number, and colocalization. In females, *Bdnf* correlated positively with total and non-primary neurite length. These patterns are broadly consistent with the established role of BDNF–TrkB signaling in neuronal survival, neurite growth, and synaptic regulation [59,60,61,62,63,64,65,66,67,68,69], while also highlighting context-dependent effects, as reduced BDNF has in some settings been associated with enhanced neurogenesis and recovery [70].

*Cask* showed stronger negative associations with neurite- and synapse-related parameters in females, in keeping with the known role of CASK in neurite and synapse biology [71,72,73]. By contrast, *Igf1* showed strong positive associations with male-biased neurite and synaptic features, consistent with the established role of IGF1 in hippocampal development, neurite extension, and synaptic maturation [74,75,76,77,78,79]. *Glul* was more strongly associated with structural responses in females, in line with its role in glutamate–glutamine cycling and hippocampal integrity [80,81]. Although *Bdnf*, *Cask*, *Igf1*, and *Glul* were selected as biologically relevant candidates, validation of only four genes is modest relative to the total number of DEGs identified. Therefore, the gene–phenotype relationships derived from this limited panel should be interpreted as exploratory and hypothesis-generating rather than comprehensive. Overall, these relationships are correlative and should be interpreted as prioritizing candidate mechanisms for future testing rather than establishing causality.

To explore possible upstream mechanisms, we combined IPA with molecular docking of the two major CNP bioactive compounds, C3G and resveratrol. Several predicted regulators, including MYC, TP53, CREB1, and HTT, are linked to neuronal function and stress responses [82,83], and many plant compounds are known to influence these pathways [84,85,86]. Because CNP contains anthocyanin-C3G and resveratrol, both of which have reported neuroprotective actions [87,88,89,90,91], we asked whether these compounds might bind the predicted regulators. Several targets, including MYC, TP53, MYCN, HTT, CREB1, MLXIPL, and HIF1A, showed favorable docking with both compounds, and these regulators are also predicted to influence *Igf1*, *Bdnf*, and *Glul*. Previous studies have linked MYC, MYCN, and TP53 to cell-cycle and apoptosis-related processes in neurodegeneration [82,92], HTT to Huntington’s disease and disrupted CREB1-mediated survival signaling [93,94], MLXIPL to neuroinflammation [95], and HIF1A to hypoxia and oxidative stress responses [96]. Together with our prior observation that CNP reduces Bax, caspase-3, and caspase-9 in peroxide-challenged cell lines [42], these findings support a working model in which C3G and resveratrol may influence stress- and survival-related regulators and thereby affect downstream genes relevant to neurite growth and synapse formation.

The docking analysis in this study was used to generate hypotheses regarding possible interactions between the major CNP bioactive compounds and predicted upstream regulatory proteins. These findings provide preliminary, hypothesis-generating insight into possible molecular mechanisms and help prioritize candidate ligand–protein interactions for future investigation, but they should not be interpreted as evidence of direct binding or target engagement in neuronal cells. To further strengthen mechanistic understanding, additional approaches such as molecular dynamics simulations and direct binding assays, including surface plasmon resonance or isothermal titration calorimetry, may be valuable in future studies.

Transcriptome enrichment also pointed to NRF2-mediated oxidative stress response, Sirtuin signaling, and neuroinflammation, consistent with prior studies showing that CNP, C3G, and resveratrol modulate these pathways in neuronal models [24,25,27,28,42]. In addition, both resveratrol and C3G have been reported to reach the brain [97,98,99,100,101,102,103,104,105]. A recent study further showed that oral C3G administration improved cognition, enhanced synaptic plasticity, and reduced neuroinflammation, apoptosis, and tau phosphorylation in APPswe mice [90]. These findings strengthen the biological plausibility of the present results. However, CNP is a multicomponent extract that also contains additional phytochemicals, and contributions from constituents other than C3G and resveratrol cannot be excluded. Therefore, although docking focused on these two compounds because of their relative abundance and biological plausibility, the present study does not attribute the observed cellular effects exclusively to them.

CNP’s major polyphenols can also interact with estrogen (ER) and androgen (AR) receptors [23,42,106]. Resveratrol is well-established for its ability to bind to and modulate estrogen receptors (ERs) [107], which is highly expressed in the hippocampus and plays a critical role in neuroprotection, synaptic plasticity, and cognitive function in both sexes [108]. C3G and its aglycone, cyanidin, have also been shown to exert estrogen receptor-modulating activity [109]. Given that ER-α and ER-β exhibit differential expression patterns between males and females, and across neuronal and glial subtypes, the phytoestrogenic components of CNP extract may evoke sex-specific effects on neuronal development. Moreover, both resveratrol and C3G have been reported to interact with androgen receptors (ARs) [110,111], which are expressed in hippocampal neurons [112]. Activation or inhibition of ARs can significantly influence gene expression programs related to neuronal growth and synapse formation in a sex-specific manner [113,114]. Thus, phytoestrogen-like and androgen-modulating actions of CNP components provide one plausible explanation for the sex differences observed here, although this remains speculative and requires direct experimental validation.

From a translational perspective, our findings suggest that CNP extract may not exert identical neurobiological effects in males and females. In males, CNP produced a more pronounced enhancement of neurite complexity and a selective increase in Syn1–Psd95 colocalization, consistent with strengthened excitatory synaptic connectivity in hippocampal neurons. In females, CNP robustly promoted neurite outgrowth without a corresponding increase in Psd95-associated puncta, suggesting that structural elaboration can occur without parallel synaptic maturation under these conditions. If similar patterns are recapitulated in vivo, such sex-dependent responses could influence how CNP-based interventions modulate hippocampal-dependent functions, resilience to neurodevelopmental or neurodegenerative insults, and the design of future neuroprotective or neurorestorative studies. These findings may also inform preclinical study design, biomarker selection, and mechanistic evaluation of plant-derived compounds. However, because the present study was conducted in primary rat hippocampal neurons in vitro and focused on early mechanistic and phenotypic endpoints rather than disease models or in vivo efficacy, these implications should be regarded as hypothesis-generating rather than as a basis for sex-specific clinical recommendations at this stage. Future sex-stratified in vivo studies will be required to determine the translational relevance of these findings. Moreover, because the present study used primary rat hippocampal cultures rather than human neurons or intact in vivo circuits, extrapolation to human neurobiology should be made cautiously.

Finally, future sex-stratified in vivo studies will be essential to validate these findings in intact brain environments and to define the dose range, cellular targets, and functional relevance of CNP. Taken together, our data indicate that CNP extract alters transcriptional programs and enhances neurite outgrowth in primary rat hippocampal neurons, with male-biased effects on Syn1–Psd95 colocalization under the present in vitro conditions. Because this study used pooled litter-derived cultures, a single working concentration for downstream assays, and limited RT-qPCR and synaptic validation, the findings should be interpreted as mechanistic and hypothesis-generating rather than definitive. Nevertheless, they support further investigation of CNP as a food-derived candidate for brain health.

## 5. Conclusions

This study provides initial evidence that CNP extract exerts sex-dependent neuroenhancing effects in primary hippocampal neurons. At non-cytotoxic concentrations, CNP altered transcriptomic profiles, enhanced neuritogenesis in both sexes, and promoted synaptogenesis more prominently in males. Gene–phenotype correlations identified *Igf1* as a candidate mediator of the male-biased response and *Glul* as a candidate mediator of female-associated structural responses, while upstream regulator and docking analyses suggested that the major CNP bioactive compounds, C3G and resveratrol, may influence transcriptional networks through sex-biased pathways.

Together, these findings improve our understanding of how CNP extract may influence neuronal development and highlight the importance of considering sex as a biological variable in nutraceutical research. Because the present study was conducted in primary rat hippocampal neurons in vitro, future sex-stratified in vivo studies and mechanistic validation will be required to determine the translational relevance of CNP for supporting brain health and enhancing neuronal plasticity.

## Figures and Tables

**Table 1:** Neurological canonical pathway associated with genes differentially expressed in the rat primary hippocampal cells treated with CNP fruit extract.

Ingenuity Canonical Pathways	*p*-Value	Number of Genes
**Male**		
EIF2 Signaling	3.98 × 10^−39^	105
Synaptogenesis-Signaling Pathway	7.94 × 10^−22^	100
Huntington’s Disease Signaling	7.94 × 10^−20^	90
Axonal Guidance Signaling	5.01 × 10^−13^	116
GABA Receptor Signaling	1.00 × 10^−11^	45
Reelin Signaling in Neurons	1.26 × 10^−11^	46
Estrogen Receptor Signaling	1.15 × 10^−10^	93
Integrin Signaling	2.57 × 10^−10^	58
Sirtuin-Signaling Pathway	1.02 × 10^−9^	71
CREB Signaling in Neurons	1.29 × 10^−6^	110
Neuroinflammation-Signaling Pathway	1.62 × 10^−6^	66
IGF-1 Signaling	5.89 × 10^−6^	29
NRF2-mediated Oxidative Stress Response	9.12 × 10^−6^	51
Synaptic Long-Term Potentiation	1.41 × 10^−5^	33
WNT/β-catenin Signaling	8.51 × 10^−5^	38
**Female**		
Synaptogenesis-Signaling Pathway	1.26 × 10^−32^	189
Huntington’s Disease Signaling	2.00 × 10^−24^	161
Axonal Guidance Signaling	6.31 × 10^−17^	230
Sirtuin-Signaling Pathway	3.16 × 10^−14^	143
IGF-1 Signaling	3.16 × 10^−11^	62
Synaptic Long-Term Potentiation	5.01 × 10^−7^	64
CREB Signaling in Neurons	7.08 × 10^−7^	225
PI3K/AKT Signaling	2.04 × 10^−6^	87
NGF Signaling	4.68 × 10^−6^	57
ERK/MAPK Signaling	4.79 × 10^−6^	91
WNT/β-catenin Signaling	2.88 × 10^−5^	74
NRF2-mediated Oxidative Stress Response	4.17 × 10^−5^	95
Apoptosis Signaling	4.47 × 10^−3^	42
TGF-β Signaling	9.77 × 10^−3^	38
Neuroinflammation-Signaling Pathway	1.10 × 10^−2^	108

The IPA software (content version 127006219, release date: 17 November 2024) predicted the neurological canonical pathway of differentially expressed genes in the rat primary hippocampal cells treated with CNP fruit extract. Statistical significance was determined using Fisher’s exact test. A *p*-value  <  0.05 was considered significant.

**Table 2:** Neurological diseases and functions associated with genes differentially expressed in the rat primary hippocampal cells treated with CNP fruit extract.

Neurological-Associated Disease and Function	*p*-Value	Number of Genes
**Male**		
Cognitive impairment	1.04 × 10^−47^	368
Huntington’s Disease	2.06 × 10^−45^	290
Alzheimer’s disease	1.51 × 10^−27^	222
Neuritogenesis	7.00 × 10^−72^	368
Potentiation of synapses	2.68 × 10^−31^	127
Migration of neurons	5.62 × 10^−18^	96
Memory	1.32 × 10^−21^	125
**Female**		
Cognitive impairment	4.03 × 10^−83^	763
Huntington’s Disease	9.01 × 10^−62^	555
Cell death of brain cells	2.13 × 10^−25^	202
Neuritogenesis	2.31 × 10^−98^	686
Development of neurons	2.31 × 10^−97^	822
Synaptic transmission	1.03 × 10^−44^	285
Memory	2.94 × 10^−32^	243

The IPA software predicted the neurological diseases and functions of differentially expressed genes in the rat primary hippocampal cells treated with CNP fruit extract. Statistical significance was determined using Fisher’s exact test. A *p*-value  <  0.05 was considered significant.

**Table 3:** Binding energies of representative upstream regulators with their known ligands, resveratrol, and cyanidin-3-glucoside (C3G).

Upstream Regulators	Ligand	Binding Energy (kcal/mol)
MYC	D347-2761 [44]	−7.33
	Resveratrol	−5.50
	C3G	−6.40
TP53	Nutlin-3a [45]	−5.10
	Resveratrol	−5.00
	C3G	−5.10
CREB1	666-15 [46]	−6.43
	Resveratrol	−6.40
	C3G	−6.70
HTT	A1ACS	−6.83
	Resveratrol	−5.37
	C3G	−6.27

Molecular docking was performed between the representative upstream regulators involved in regulating DEGs in male and female rat primary hippocampal cells and their known ligands, including resveratrol and cyanidin-3-glucoside (C3G), using BIOVIA Discovery Studio Visualizer 2020 and AutoDockTools-1.5.6 software. The mean binding-free energy values for each pair of upstream regulators and their ligands were calculated from triplicate experiments. More negative binding-energy values indicate stronger predicted binding affinity. Detailed docking results for all 10 upstream regulators, including PDB IDs and amino acid interactions, are provided in Supplementary Table S6.

## Data Availability

The datasets analyzed during the current study are available in the GEO DataSets repository, [https://www.ncbi.nlm.nih.gov/geo/query/acc.cgi?acc=GSE284729], accessed date: 2 May 2024, and are available from the corresponding author on reasonable request.

## Supplementary Materials

The following supporting information can be downloaded at https://www.mdpi.com/article/10.3390/nu18081200/s1, Table S1: List of primers for qRT-PCR analyses. Table S2: Full list of differentially expressed genes (DEGs) identified in male and female rat primary hippocampal cells in response to CNP extract, together with baseline male–female control DEGs. Table S3: Overlap of differentially expressed genes among CNP-treated males versus male controls, CNP-treated females versus female controls, and untreated male versus untreated female control hippocampal cells. Table S4: Mean ± SEM values for all quantitative measures presented in RT-qPCR, neuritogenesis, and synaptogenesis experiment for each sex and treatment group. Table S5: Phytochemical compositions of ripe *Cleistocalyx nervosum* var. *paniala* fruit. Table S6: Molecular docking analysis of the top 10 significant upstream regulators of differentially expressed genes (DEGs) in male and female primary hippocampal cells treated with CNP fruit extract. References [115,116,117,118,119,120,121,122,123] are cited in the supplementary materials. Figure S1: Complete description of node and edge types used in the Ingenuity Pathway Analysis (IPA) software (content version 127006219, release date: 2024 November 17). Figure S2: Overlap of differentially expressed genes (DEGs) among male and female CNP responses and baseline sex differences. Figure S3: The 2D molecular docking interaction diagrams of the top 10 significant upstream regulators with their known ligands, resveratrol, and cyanidin-3-glucoside (C3G).
