# Supplementary material for: Neurological Effects of Cleistocalyx nervosum var. paniala Berry on Hippocampal Transcriptome, Neuritogenesis, and Synaptogenesis
Source: Nutrients. 2026 Apr 10;18(8):1200. doi: 10.3390/nu18081200 (PMC13119000; doi:10.3390/nu18081200)

Figure S1: Complete description of node and edge types used in the Ingenuity Pathway Analysis (IPA) software.

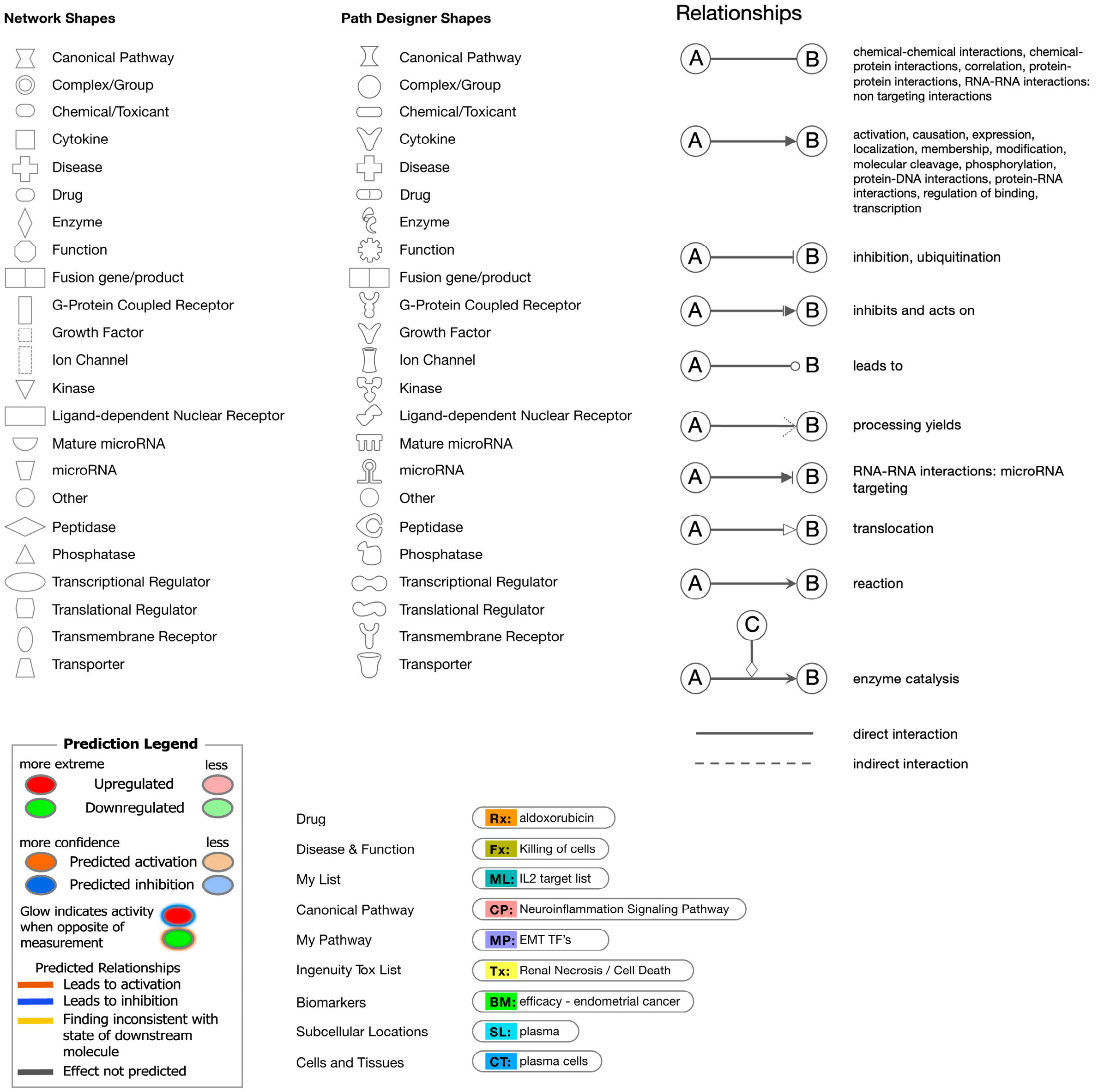

Supplement: Supplementary file 1 [file nutrients-18-01200-s001.zip › Figure S1.pdf]
