# Supplementary material for: Neurological Effects of Cleistocalyx nervosum var. paniala Berry on Hippocampal Transcriptome, Neuritogenesis, and Synaptogenesis
Source: Nutrients. 2026 Apr 10;18(8):1200. doi: 10.3390/nu18081200 (PMC13119000; doi:10.3390/nu18081200)

Figure S2: Overlap of differentially expressed genes (DEGs) among male and female CNP responses and baseline sex differences. Venn diagram showing the overlap among DEGs identified in CNP-treated males versus male controls (Group A: CNP10_M vs. CNP0_M), CNP-treated females versus female controls (Group B: CNP10_F vs. CNP0_F), and control males versus control females (Group C: CNP0_M vs. CNP0_F). Numbers represent the number of genes unique to each comparison and shared among comparisons. This analysis was used to determine the extent to which CNP-responsive DEGs in males and females overlapped with baseline sex-differential genes.

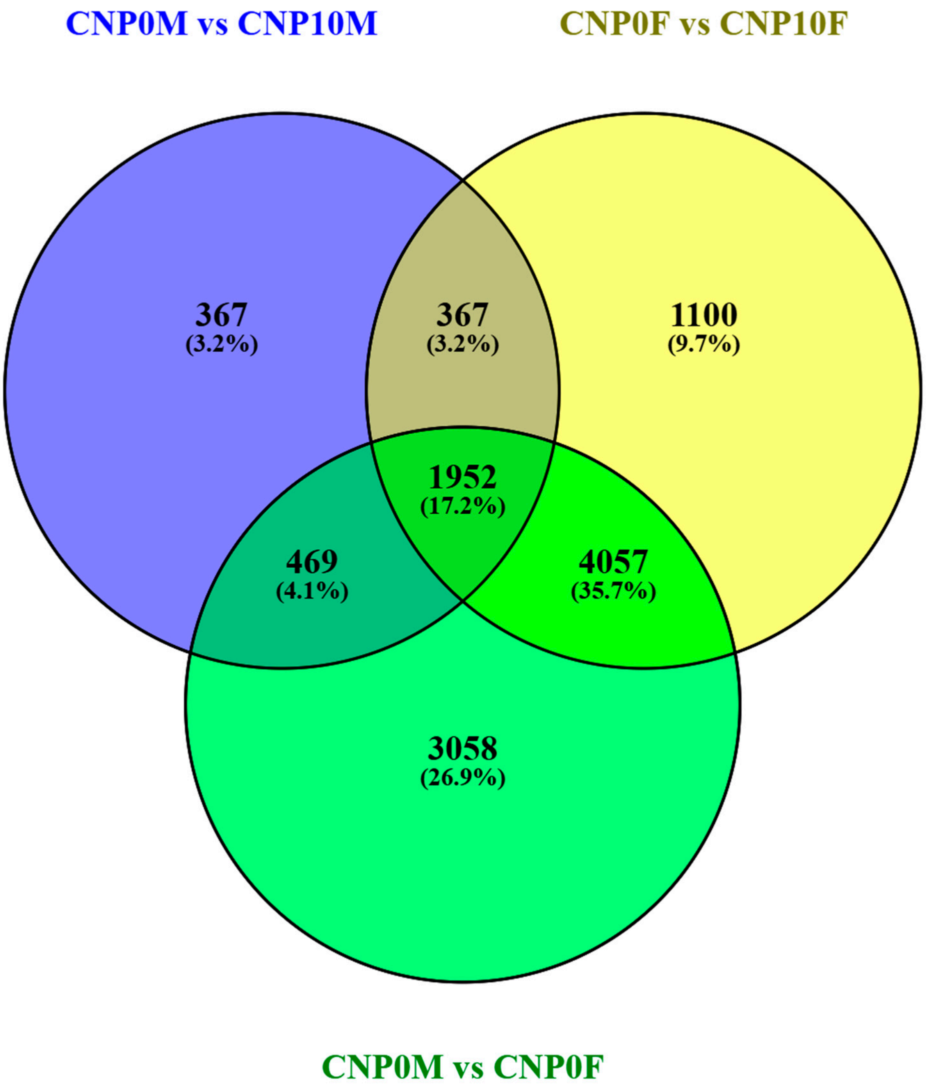

Supplement: Supplementary file 1 [file nutrients-18-01200-s001.zip › Figure S2.pdf]
