# Supplementary material for: Neurological Effects of Cleistocalyx nervosum var. paniala Berry on Hippocampal Transcriptome, Neuritogenesis, and Synaptogenesis
Source: Nutrients. 2026 Apr 10;18(8):1200. doi: 10.3390/nu18081200 (PMC13119000; doi:10.3390/nu18081200)
Supplement: Supplementary file 1 [file nutrients-18-01200-s001.zip › Figure S3.pdf]

Figure S3: The 2D molecular docking interaction diagrams of the top 10 significant upstream regulators with their known ligands, resveratrol, and cyanidin-3-glucoside (C3G). The HIF1A, RB1, MYCN, MLXIPL, CTNNB1, HNF4A, TEAD1, YAP1, FOS, NFE2L2 significant upstream regulators of male or female DEGs in rat primary hippocampal cells treated with CNP berry extract. Dashed lines are color-coded to indicate interaction types between the ligand and protein residues: bright green = conventional hydrogen bond; light green = carbon–hydrogen interaction; yellow-green = π–lone-pair interaction; pink = hydrophobic/π-alkyl interaction; deep pink = π–π stacking interaction; purple = π–sigma interaction; orange = π–cation/anion interaction; yellow-orange = π–sulfur interaction; red residue circle = unfavorable interaction site; red dashed line = unfavorable contact. Pale or lightly shaded residue circles without an interaction line indicate van der Waals contacts, and a blue halo surrounding residues represents the solvent-accessible surface. Residue circles are colored according to the most favorable interaction present at that site when multiple interaction types occur along different dashed lines.

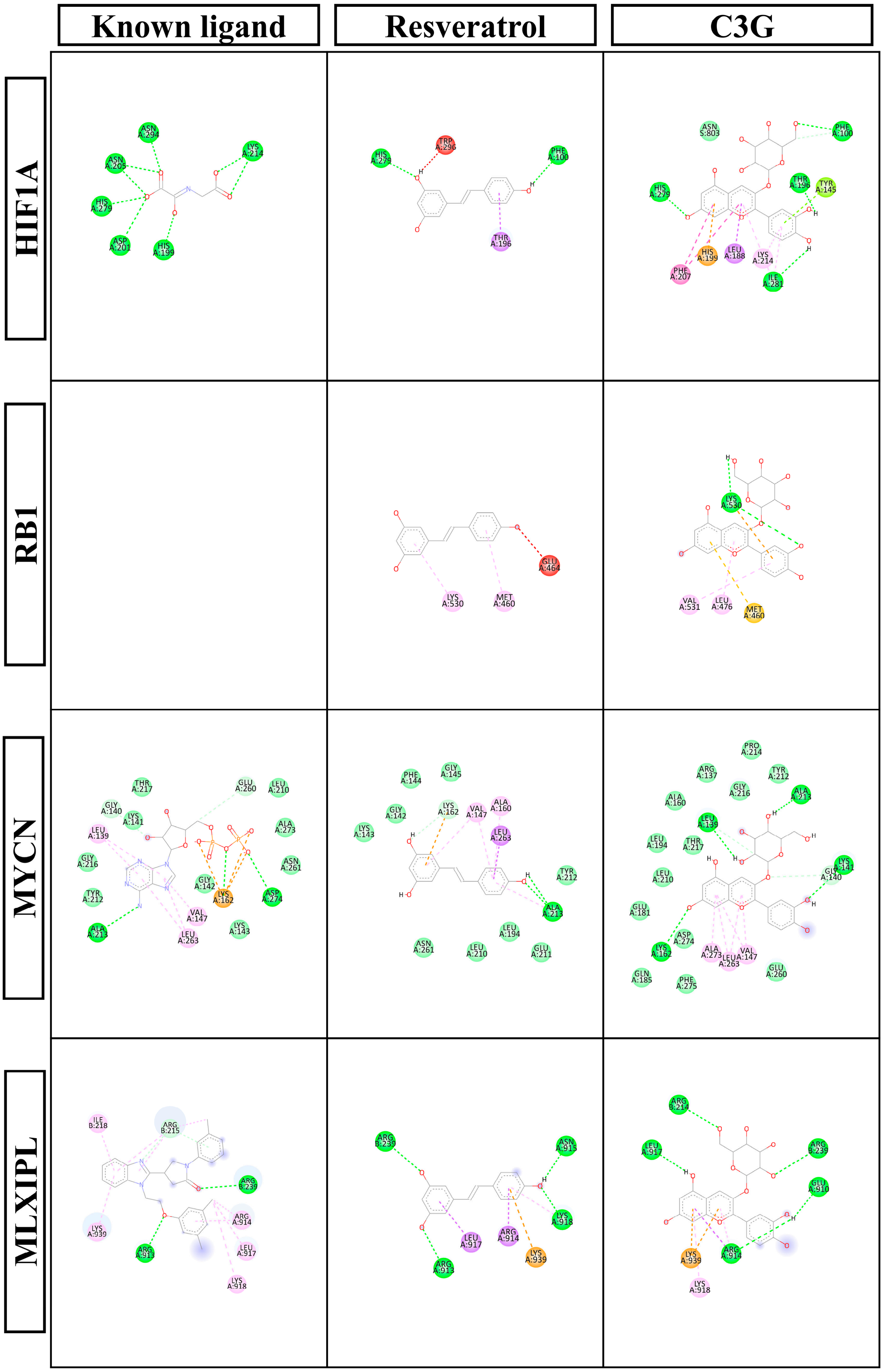
Known ligand
Resveratrol
C3G
HIF1A
RB1
MYCN
MLXIPL

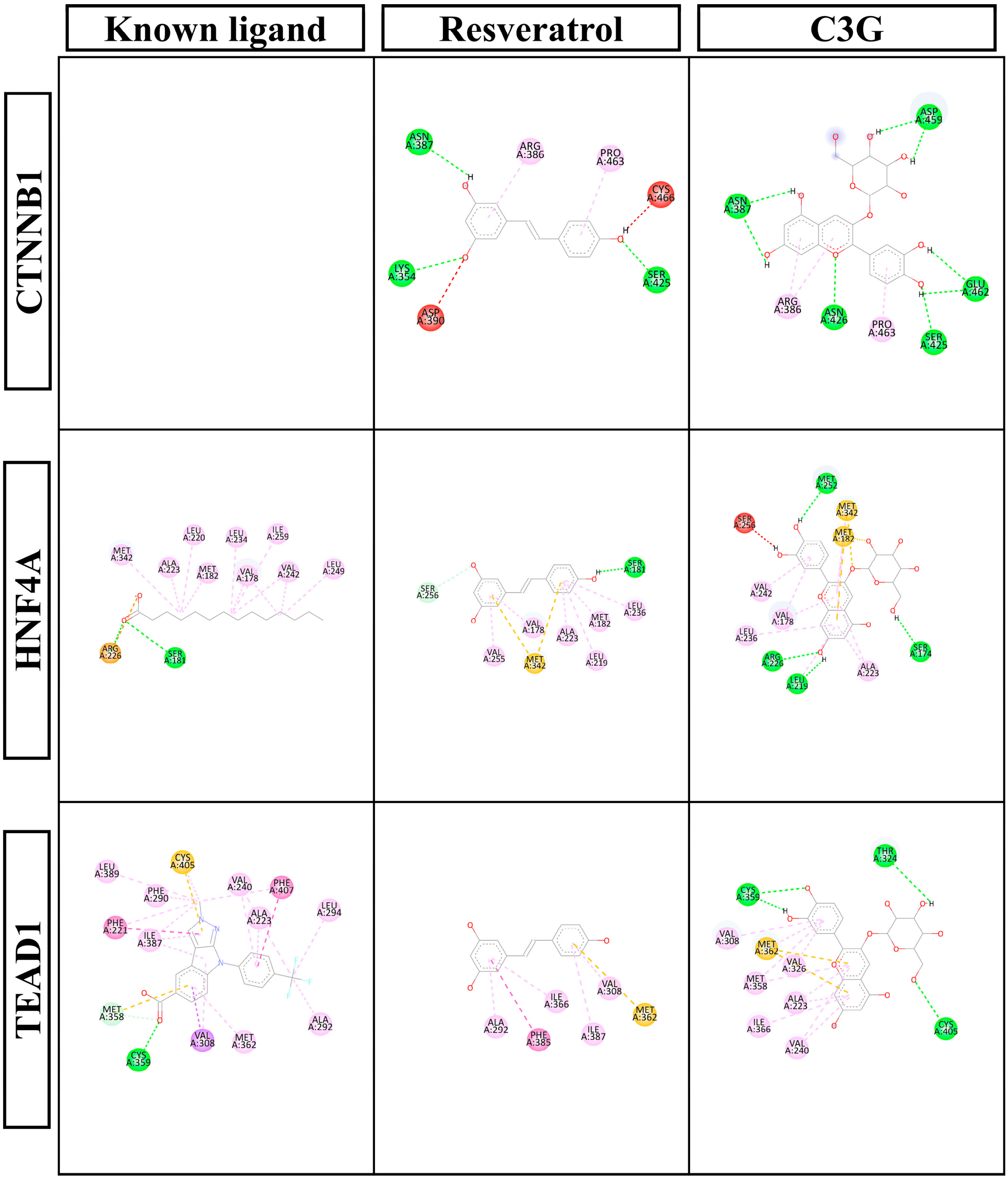

Known ligand
Resveratrol
C3G
CTNNB1
HNF4A
TEAD1

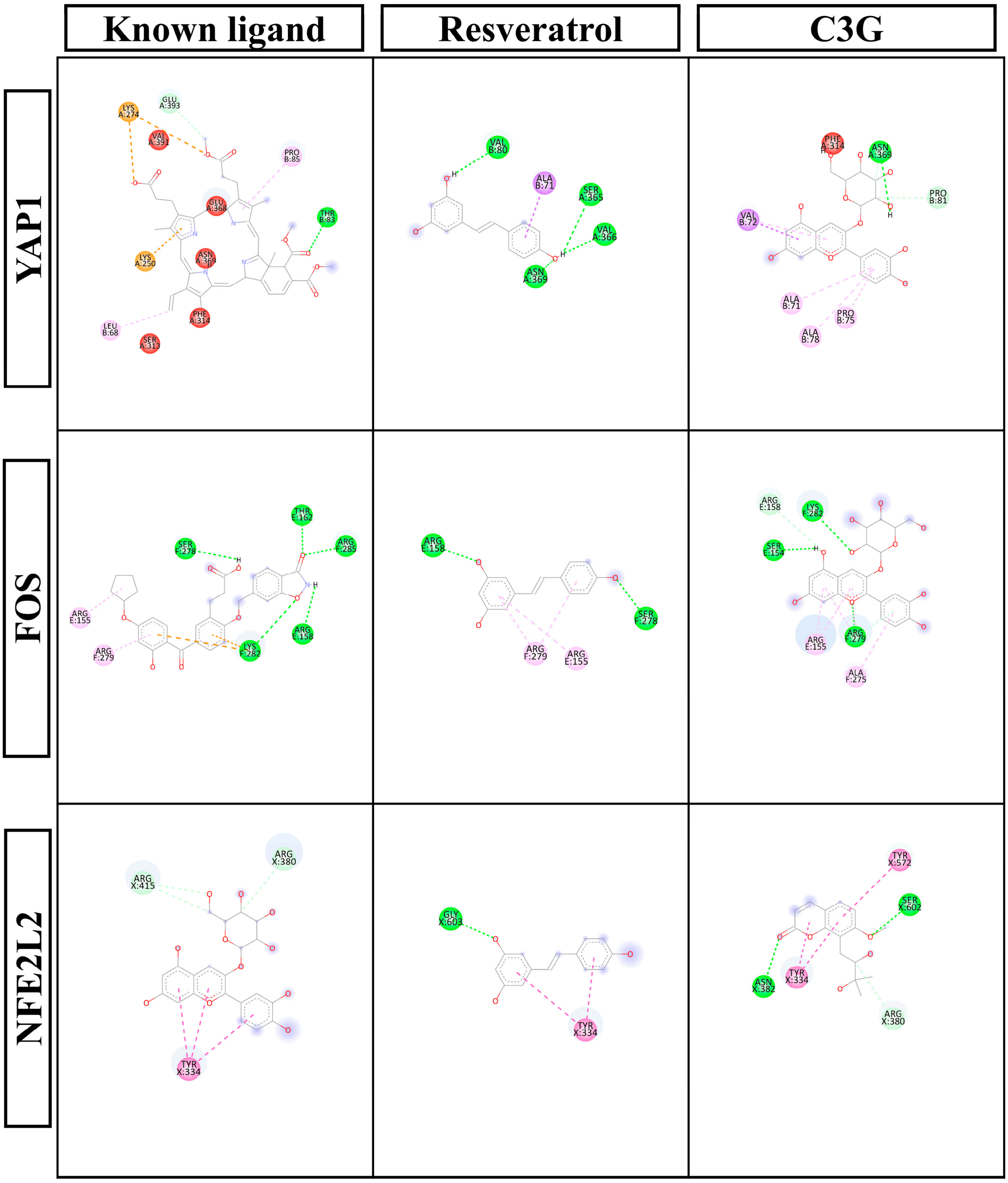
Known ligand
Resveratrol
C3G
YAP1
FOS
NFE2L2
