# Supplementary material for: Neurological Effects of Cleistocalyx nervosum var. paniala Berry on Hippocampal Transcriptome, Neuritogenesis, and Synaptogenesis
Source: Nutrients. 2026 Apr 10;18(8):1200. doi: 10.3390/nu18081200 (PMC13119000; doi:10.3390/nu18081200)
Supplement: Supplementary file 1 [file nutrients-18-01200-s001.zip › Table S1.pdf]

Table S1. List of primers for qRT-PCR analyses.

Genes	Nucleotide sequences (5′ to 3′)
Rat *Igf1* forward	ATAGAGCCTGCGCAATCGAA
Rat *Igf1* reverse	TAGCCTGTGGGCTTGTTGAA
Rat *Bdnf* forward	TACCTGGATGCCGCAAACAT
Rat *Bdnf* reverse	TGGCCTTTTGATACCGGGAC
Rat *Cask* forward	TGCTTAGGGAAATGCGAGGG
Rat *Cask* reverse	TGGTTGATGGCAAGTCCGAA
Rat *Glul* forward	TGCGAAGACTTTGGGGTGAT
Rat *Glul* reverse	TCAATGGCCTCCTCAATGCA
Rat *Rn18s* forward	CTGGATACCGCAGCTAGGAA
Rat *Rn18s* reverse	GAATTTCACCTCTAGCGGCG
