# Supplementary material for: Neurological Effects of Cleistocalyx nervosum var. paniala Berry on Hippocampal Transcriptome, Neuritogenesis, and Synaptogenesis
Source: Nutrients. 2026 Apr 10;18(8):1200. doi: 10.3390/nu18081200 (PMC13119000; doi:10.3390/nu18081200)
Supplement: Supplementary file 1 [file nutrients-18-01200-s001.zip › Table S4.pdf]

Table S4. Mean ± SEM values for all quantitative measures presented in RT-qPCR, neuritogenesis, and synaptogenesis experiment for each sex and treatment group.

Relative mRNA expression

	**Control_M**		**CNP10_M**		**Control_F**		**CNP10_F**	
	**Mean**	**SEM.**	**Mean**	**SEM.**	**Mean**	**SEM.**	**Mean**	**SEM.**
Bdnf	1.00	0.20	0.33	0.01	1.00	0.18	1.44	0.31
Cask	1.00	0.11	0.35	0.05	1.00	0.33	1.25	0.26
Igf1	1.00	0.08	3.29	0.45	1.00	0.39	1.72	0.39
Glul	1.00	0.69	0.72	0.28	1.00	0.02	2.48	0.13

Neuritogenesis parameters

Total neurite length (µm)	Mean	SEM.
Control_M	382.34	15.18
CNP10_M	486.80	20.57
Control_F	278.01	11.86
CNP10_F	388.11	15.31

Average neurite length	Mean	SEM.
Control_M	43.19	1.93
CNP10_M	43.32	1.61
Control_F	36.82	1.23
CNP10_F	38.41	1.85

Number of neurite branches	Mean	SEM.
Control_M	11.47	0.44
CNP10_M	16.69	0.77
Control_F	9.47	0.40
CNP10_F	14.44	0.61

Primary neurite length (µm)	Mean	SEM.
Control_M	43.73	2.43
CNP10_M	42.17	1.92
Control_F	35.19	1.23
CNP10_F	38.91	2.17

Non-primary neurite length (µm)	Mean	SEM.
Control_M	34.91	1.57
CNP10_M	40.49	1.73
Control_F	27.70	1.66
CNP10_F	32.97	1.47

Number of primary neurites	Mean	SEM.
Control_M	4.06	0.11
CNP10_M	4.82	0.15
Control_F	3.54	0.11
CNP10_F	4.31	0.12

Number of non-primary neurites	Mean	SEM.
Control_M	7.41	0.39
CNP10_M	11.87	0.70
Control_F	5.59	0.35
CNP10_F	10.12	0.56

Sholl analysis

Number of intersections	Control_M		CNP10_M		Control_F		CNP10_F	
Distance from soma (μm)	Mean	SEM.	Mean	SEM.	Mean	SEM.	Mean	SEM.
0	0.00	0.00	0.00	0.00	0.00	0.00	0.00	0.00
10	4.76	0.12	5.42	0.15	4.40	0.11	4.98	0.12
20	4.65	0.13	5.59	0.20	4.57	0.13	5.23	0.16
30	4.88	0.17	6.18	0.24	4.54	0.16	5.34	0.18
40	4.61	0.17	6.15	0.24	3.86	0.16	5.14	0.19
50	4.13	0.18	5.76	0.25	2.96	0.16	4.38	0.20
60	3.62	0.19	4.92	0.24	2.42	0.17	3.66	0.21
70	2.99	0.19	4.27	0.27	1.83	0.16	2.98	0.22
80	2.51	0.18	3.63	0.26	1.41	0.15	2.51	0.22
90	2.13	0.18	3.22	0.28	1.04	0.13	2.07	0.20
100	1.70	0.16	2.86	0.29	0.83	0.12	1.56	0.17
110	1.45	0.16	2.54	0.28	0.68	0.11	1.35	0.16
120	1.23	0.16	2.25	0.26	0.58	0.10	1.24	0.16
130	1.01	0.13	1.96	0.24	0.49	0.09	1.04	0.14
140	0.84	0.13	1.77	0.23	0.36	0.07	0.84	0.12
150	0.72	0.12	1.46	0.21	0.29	0.06	0.71	0.11
160	0.62	0.11	1.21	0.19	0.26	0.06	0.61	0.10
170	0.54	0.09	1.01	0.16	0.20	0.05	0.56	0.10
180	0.47	0.08	0.82	0.15	0.18	0.04	0.51	0.09
190	0.33	0.07	0.68	0.12	0.17	0.04	0.39	0.08
200	0.25	0.06	0.59	0.12	0.14	0.04	0.30	0.06
210	0.20	0.05	0.54	0.12	0.12	0.04	0.24	0.05
220	0.14	0.04	0.46	0.11	0.09	0.03	0.15	0.04
230	0.11	0.04	0.37	0.09	0.07	0.03	0.11	0.03
240	0.08	0.03	0.29	0.07	0.06	0.03	0.07	0.02
250	0.08	0.03	0.23	0.07	0.04	0.02	0.07	0.02
260	0.08	0.03	0.18	0.05	0.03	0.02	0.05	0.02
270	0.06	0.03	0.11	0.04	0.02	0.01	0.03	0.01
280	0.04	0.02	0.10	0.04	0.00	0.00	0.02	0.01
290	0.03	0.02	0.09	0.04	0.00	0.00	0.01	0.01
300	0.03	0.02	0.07	0.03	0.00	0.00	0.01	0.01
310	0.02	0.01	0.07	0.03	0.00	0.00	0.01	0.01
320	0.02	0.01	0.05	0.02	0.00	0.00	0.01	0.01
330	0.02	0.01	0.04	0.02	0.00	0.00	0.01	0.01
340	0.01	0.01	0.03	0.02	0.00	0.00	0.00	0.00
350	0.01	0.01	0.03	0.02	0.00	0.00	0.00	0.00
360	0.01	0.01	0.02	0.02	0.00	0.00	0.00	0.00
370	0.01	0.01	0.02	0.02	0.00	0.00	0.00	0.00
380	0.00	0.00	0.02	0.02	0.01	0.01	0.00	0.00
390	0.00	0.00	0.00	0.00	0.00	0.00	0.00	0.00
400	0.00	0.00	0.00	0.00	0.00	0.00	0.00	0.00

Synaptogenesis parameters

Pearson's coefficient	Mean	SEM.
Control_M	0.47	0.05
CNP10_M	0.47	0.04
Control_F	0.48	0.05
CNP10_F	0.48	0.05

Percentage of colocalization	Mean	SEM.
Control_M	21.84	2.60
CNP10_M	30.18	3.18
Control_F	33.26	3.48
CNP10_F	26.56	3.02

Total Syn1 puncta per 100 µm	Mean	SEM.
Control_M	71.89	8.07
CNP10_M	77.51	8.28
Control_F	87.52	10.35
CNP10_F	69.55	7.73

Total Psd95 puncta per 100 µm	Mean	SEM.
Control_M	198.07	23.04
CNP10_M	248.20	26.86
Control_F	287.33	34.83
CNP10_F	218.14	24.57
