# Supplementary material for: Neurological Effects of Cleistocalyx nervosum var. paniala Berry on Hippocampal Transcriptome, Neuritogenesis, and Synaptogenesis
Source: Nutrients. 2026 Apr 10;18(8):1200. doi: 10.3390/nu18081200 (PMC13119000; doi:10.3390/nu18081200)
Supplement: Supplementary file 1 [file nutrients-18-01200-s001.zip › Table S5.pdf]

Table S5. Phytochemical compositions of ripe *Cleistocalyx nervosum* var. *paniala* fruit

Phytochemical	Content (per 100 g dry weight)
Apigenin	ND
Ferulic acid	65.13 ± 0.91 μg
Hesperetin	ND
Kaempferol	ND
Luteolin	ND
Myricetin	ND
Naringenin	ND
Quercetin	6,016.76 ± 8.70 μg
Cyanidin-3-glucoside	163.25 ± 0.02 mg
Resveratrol	18.42 ± 0.09 mg
Lutein	634.19 ± 4.31 μg
Zeaxanthin	ND
Beta-cryptoxanthin	ND
Lycopene	ND
Alpha-carotene	ND
Beta-carotene	125.96 ± 2.19 μg

Values are mean ± SD (n = 3), Not detected (ND)

Note: analyzed by the Laboratory Analytical Service of Institute of Nutrition, Mahidol University, in accordance with the internationally recognized ISO/IEC 17025:2005 and 2017 standards.
