# Supplementary material for: Neurological Effects of Cleistocalyx nervosum var. paniala Berry on Hippocampal Transcriptome, Neuritogenesis, and Synaptogenesis
Source: Nutrients. 2026 Apr 10;18(8):1200. doi: 10.3390/nu18081200 (PMC13119000; doi:10.3390/nu18081200)
Supplement: Supplementary file 1 [file nutrients-18-01200-s001.zip › Table S6.pdf]

Table S6. Molecular docking analysis of the top 10 significant upstream regulators of differentially expressed genes (DEGs) in male and female primary hippocampal cells treated with CNP fruit extract.

Upstream regulators	PDB ID	Ligand	Binding Energy (kcal/mol)	Inhibition Constant (μM)	Amino Acid Interaction		
					Hydrogen Bond	Hydrophobic Bond	Others
CREB1	5ZK1 (A: Cyclic AMP-responsive element-binding protein 1 (CREB1) and B: CREB-regulated transcription coactivator 2	666-15 [1]	-6.43	19.22	A: GLU299, ALA296<br>B: LYS21	A: ALA296(2), LYS292<br>B: ARG20, PHE22, LYS21(2)	A: -<br>B: LYS21
		Resveratrol	-6.40	20.34	A: LYS303(2)<br>B: LYS21	A: GLU299, LYS303<br>B: PHE22, LYS21	A: -<br>B: -
		C3G	-6.70	12.26	A: SER300, LYS303(2), ALA296, LYS21<br>B: SER23	A: GLU299(2), LYS303<br>B: PHE22, LYS21(2)	A: LYS21<br>B: -
CTNNB1	7AR4	E-cadherin [2]	-16.60	6.77E-07	LYS435, LYS508, GLU462(2), LYS354, ASN426(2), ASN430, ASP390	CYS429(2), ARG386	ARG469(2), HIS470, ARG474
		Resveratrol	-5.47	98.29	LYS354, SER425, ASN387	ARG386, PRO463	-
		C3G	-6.37	21.51	SER425, ASN426, ASP459(2), GLU462(2), ASN387(2)	ARG386(2), PRO463	-
FOS	1FOS (A: P55-C-FOS proto-oncogene protein (FOS) and B: C-JUN proto-oncogene protein)	T-5224 [3]	-6.23	26.94	A: THR162, ARG158<br>B: LYS282, ARG285, SER278	A: ARG155<br>B: ARG279, LYS282	A: -<br>B: LYS282(2)
		Resveratrol	-4.57	449.02	A: ARG158<br>B: SER278	A: ARG155<br>B: ARG279(2)	A: -<br>B: -
		C3G	-5.13	172.53	A: SER154, ARG158<br>B: ARG279(2), LYS282(2)	A: ARG155(2)<br>B: ARG279(3), ALA275	A: -<br>B: -

Upstream regulators	PDB ID	Ligand	Binding Energy (kcal/mol)	Inhibition Constant (µM)	Amino Acid Interaction		
					Hydrogen Bond	Hydrophobic Bond	Others
HIF1A	1H2K (A: Factor Inhibiting (HIF1) and B: HYPOXIA-INDUCIBLE FACTOR 1 ALPHA (HIF1A))	N-oxaloylglycine [4]	-5.33	123.09	A: HIS199, ASN205(2), LYS214(4), HIS279, ASN294, ASP201 B: -	A: - B: -	A: - B: -
		Resveratrol	-6.00	39.95	A: HIS279, PHE100 B: -	A: THR196 B: -	A: - B: -
		C3G	-6.57	15.35	A: PHE100(2), HIS279, THR196, ILE281 B: -	A: LEU188, PHE207(2), HIS199, ILE281(2), LYS214 B: -	A: HIS199, TYR145 B: -
HNF4A	4IQR	Myristic acid [5]	-5.57	83.02	SER181, ARG226	VAL178(2), ALA223, LEU234,VAL242(2), ILE259, MET182, LEU220, MET342, LEU249	ARG226
		Resveratrol	-5.83	52.93	SER181, SER256	VAL178, VAL255, MET182, LEU219, ALA223, LEU236	MET342(2)
		C3G	-2.70	10487.76	ARG226, SER174, MET252, LEU219	VAL178(2), MET182(2), ALA223(2), MET342, LEU219, LEU236, VAL242	MET182, MET342(2)
HTT	8VLX	A1ACS	-6.83	9.79	ILE2595, ALA2587	MET2588, PRO2417, ARG2592, ALA2587, CYS2998	GLU2584, MET2588
		Resveratrol	-5.37	116.36	-	PRO2417, LEU2600	-

Upstream regulators	PDB ID	Ligand	Binding Energy (kcal/mol)	Inhibition Constant (µM)	Amino Acid Interaction		
					Hydrogen Bond	Hydrophobic Bond	Others
		C3G	-6.27	25.47	SER2590, ARG2592(2), MET2588, ALA2587, ILE2595	ALA2413, PRO2417, ARG2592	-
MLXIPL	6YGJ (A: 14-3-3 protein beta/alpha and B: Carbohydrate-responsive element-binding protein (MLXIPL)	STK631094 [6]	-7.50	3.18	A: LYS51(2), ARG58(2), TYR130, ASN175, ARG129(3) B: ARG128(3)	A: LEU218 B: TRP127(2), ILE120	A: LYS51(2), ARG58(2), ASP126, ARG129(3) , GLU182 B: ARG128(2)
		Resveratrol	-6.43	19.22	A: ARG129 B: ARG128	A: ILE219, LEU222 B: -	A: LYS51 B: ARG128
		C3G	-8.97	0.27	A: ARG58, ARG129, TYR130(2) B: -	A: LEU222, LEU218 B: ILE120(2)	A: - B: -
MYC	1NKP (A: Myc proto-oncogene protein (MYC) and B: Max protein)	D347-2761 [7]	-7.33	4.21	A: ARG913 B: ARG239, ARG215(2)	A: ARG914(2), LEU917, LYS918, LYS939 B: ARG215(4), ILE218	A: - B: -
		Resveratrol	-5.50	92.91	A: ARG913(2), LYS918, ASN915 B: ARG239	A: ARG914, LEU917, LYS918 B: -	A: LYS939 B: -
		C3G	-6.40	20.34	A: GLU910, ARG914, LEU917 B: ARG214, ARG239	A: ARG914(2), LYS918 B: -	A: LYS939(2) B: -
MYCN	5G1X (A: N-MYC proto-oncogene protein (MYCN) and B: Aurora kinase A)	ADP [8]	-6.73	11.59	A: LYS162, ASP274, ALA213, GLY140, GLU260 B: -	A: LEU139(2), VAL147, LEU263(2) B: -	A: LYS162(3) B: -
		Resveratrol	-7.30	4.45	A: ALA213(2), LYS162 B: -	A: LEU263, VAL147(2), LA160, ALA213	A: LYS162 B: -

Upstream regulators	PDB ID	Ligand	Binding Energy (kcal/mol)	Inhibition Constant (µM)	Amino Acid Interaction		
					Hydrogen Bond	Hydrophobic Bond	Others
						B: -	
		C3G	-7.33	4.21	A: -<br>B: LYS141, LYS162, ALA213, LEU139(2), GLY140	A: -<br>B: VAL147(2), LEU263(2), ALA273	A: -<br>B: -
NFE2L2	2FLU	Meranzin Hydrate [9]	-6.30	24.08	ASN382(2), SER602, ARG380	TYR334(2), TYR572	-
		Resveratrol	-5.60	78.48	GLY603	TYR334(2)	-
		C3G	-4.40	594.90	ARG415(2), ARG380	TYR334(3)	-
TP53	4HFZ	Nutlin-3a [10]	-5.10	182.51	-	LEU22(2), PHE19, LEU25	-
		Resveratrol	-5.00	216.07	-	LEU22	-
		C3G	-5.10	182.51	LEU25, LEU26	LEU26(3), LEU22	-
RB1	1N4M	No known ligand	-	-	-	-	-
		Resveratrol	-6.17	30.15	-	LYS530, MET460	-
		C3G	-7.13	5.90	LYS530(2)	LYS530(2), LEU476, VAL531	LYS530, MET460
TEAD1	7ZJP	MSC-4106 [11]	-8.00	1.37	CYS359, MET358	VAL308(2), PHE407, PHE221, ALA292, ILE387(3), LEU389, CYS405, VAL240(2), LEU294, PHE221, PHE290, PHE407, MET362, ALA223	MET358, CYS405
		Resveratrol	-7.33	4.21	-	PHE385, ALA292, ILE366, VAL308, ILE387	MET362

Upstream regulators	PDB ID	Ligand	Binding Energy (kcal/mol)	Inhibition Constant (μM)	Amino Acid Interaction		
					Hydrogen Bond	Hydrophobic Bond	Others
		C3G	-2.10	28875.60	CYS359(2), CYS405, THR324	ALA223(2), VAL240(2), MET358(2), ILE366, VAL308, VAL326, MET362	MET362(2)
YAP1	3KYS (A: Transcriptional enhancer factor TEF-1 and B: 65 kDa Yes-associated protein (YAP1))	Verteporfin [12]	-6.13	31.90	A: GLU393 B: THR83	A: LYS250 B: LEU68, PRO85	A: LYS274(2), LYS250 B: -
		Resveratrol	-4.77	320.37	A: ASN369, SER365, VAL366 B: VAL80	A: - B: ALA71	A: - B: -
		C3G	-5.70	66.29	A: ASN369 B: PRO81	A: - B: VAL72(2), ALA71, PRO75, ALA78	A: LYS530, MET460 B: -

Molecular docking was performed between the top upstream regulators involved in regulating DEGs in male and female rat primary hippocampal cells and their known ligands, including resveratrol and cyanidin-3-glucoside (C3G) using Discovery Studio 2019 and Autodock 4.2 software. The mean binding free energy values for each pair of upstream regulators and their ligands were calculated from triplicate experiments. Amino acid interactions were determined and displayed based on the interaction between the ligand and molecule A or molecule B if the upstream regulators existed in a complex form as deposited in the RCSB Protein Data Bank database.

1. Li, B.X.; Gardner, R.; Xue, C.; Qian, D.Z.; Xie, F.; Thomas, G.; Kazmierczak, S.C.; Habecker, B.A.; Xiao, X. Systemic Inhibition of CREB is Well-tolerated in vivo. *Scientific reports* **2016**, *6*, 34513, doi:https://10.1038/srep34513.
2. Wendt, M.; Bellavita, R.; Gerber, A.; Efrém, N.-L.; van Ramshorst, T.; Pearce, N.M.; Davey, P.R.J.; Everard, I.; Vazquez-Chantada, M.; Chiarparin, E.; et al. Bicyclic β-Sheet Mimetics that Target the Transcriptional Coactivator β-Catenin and Inhibit Wnt Signaling. **2021**, *60*, 13937-13944, doi:https://doi.org/10.1002/anie.202102082.
3. Ishida, M.; Ueki, M.; Morishita, J.; Ueno, M.; Shiozawa, S.; Maekawa, N. T-5224, a selective inhibitor of c-Fos/activator protein-1, improves survival by inhibiting serum high mobility group box-1 in lethal lipopolysaccharide-induced acute kidney injury model. *Journal of Intensive Care* **2015**, *3*, 49, doi:http://10.1186/s40560-015-0115-2.
4. Elkins, J.M.; Hewitson, K.S.; McNeill, L.A.; Seibel, J.F.; Schlemminger, I.; Pugh, C.W.; Ratcliffe, P.J.; Schofield, C.J. Structure of Factor-inhibiting Hypoxia-inducible Factor (HIF) Reveals Mechanism of Oxidative Modification of HIF-1alpha. *Journal of Biological Chemistry* **2003**, *278*, 1802-1806, doi:https://10.1074/jbc.C200644200.
5. Chandra, V.; Huang, P.; Potluri, N.; Wu, D.; Kim, Y.; Rastinejad, F. Multidomain integration in the structure of the HNF-4α nuclear receptor complex. *Nature* **2013**, *495*, 394-398, doi:https://10.1038/nature11966.

6. Sijbesma, E.; Visser, E.; Plitzko, K.; Thiel, P.; Milroy, L.-G.; Kaiser, M.; Brunsveld, L.; Ottmann, C. Structure-based evolution of a promiscuous inhibitor to a selective stabilizer of protein–protein interactions. *Nature Communications* **2020**, *11*, 3954, doi:https://10.1038/s41467-020-17741-0.
7. Yao, R.; Zhang, M.; Zhou, J.; Liu, L.; Zhang, Y.; Gao, J.; Xu, K. Novel dual-targeting c-Myc inhibitor D347-2761 represses myeloma growth via blocking c-Myc/Max heterodimerization and disturbing its stability. *Cell Communication and Signaling* **2022**, *20*, 73, doi:https://10.1186/s12964-022-00868-6.
8. Richards, M.W.; Burgess, S.G.; Poon, E.; Carstensen, A.; Eilers, M.; Chesler, L.; Bayliss, R. Structural basis of N-Myc binding by Aurora-A and its destabilization by kinase inhibitors. **2016**, *113*, 13726-13731, doi:https://10.1073/pnas.1610626113.
9. Brogi, S.; Guarino, I.; Flori, L.; Sirous, H.; Calderone, V. In Silico Identification of Natural Products and World-Approved Drugs Targeting the KEAP1/NRF2 Pathway Endowed with Potential Antioxidant Profile. *Computation* **2023**, *11*, 255, doi:https://10.3390/computation11120255.
10. Anil, B.; Riedinger, C.; Endicott, J.A.; Noble, M.E.M. The structure of an MDM2-Nutlin-3a complex solved by the use of a validated MDM2 surface-entropy reduction mutant. *Acta Crystallographica Section D* **2013**, *69*, 1358-1366, doi:https://10.1107/S0907444913004459.
11. Heinrich, T.; Peterson, C.; Schneider, R.; Garg, S.; Schwarz, D.; Gunera, J.; Seshire, A.; Kötzner, L.; Schlesiger, S.; Musil, D.; et al. Optimization of TEAD P-Site Binding Fragment Hit into In Vivo Active Lead MSC-4106. *Journal of Medicinal Chemistry* **2022**, *65*, 9206-9229, doi:https://10.1021/acs.jmedchem.2c00403.
12. Wang, C.; Zhu, X.; Feng, W.; Yu, Y.; Jeong, K.; Guo, W.; Lu, Y.; Mills, G.B. Verteporfin inhibits YAP function through up-regulating 14-3-3σ sequestering YAP in the cytoplasm. *Am J Cancer Res* **2016**, *6*, 27-37.
